# On the electron pairing mechanism of copper-oxide high temperature superconductivity

**DOI:** 10.1073/pnas.2207449119

**Published:** 2022-09-06

**Authors:** Shane M. O’Mahony, Wangping Ren, Weijiong Chen, Yi Xue Chong, Xiaolong Liu, H. Eisaki, S. Uchida, M. H. Hamidian, J. C. Séamus Davis

**Affiliations:** ^a^Department of Physics, University College Cork, Cork T12 R5C, Ireland;; ^b^Clarendon Laboratory, University of Oxford, Oxford OX1 3PU, United Kingdom;; ^c^Department of Physics, Cornell University, Ithaca, NY 14850;; ^d^Kavli Institute for Nanoscale Science, Cornell University, Ithaca, NY 14853;; ^e^National Institute of Advanced Industrial Science and Technology, Tsukuba, Ibaraki 305-8568, Japan;; ^f^Department of Physics, The University of Tokyo, Bunkyo, Tokyo 113-0011, Japan;; ^g^Max-Planck Institute for Chemical Physics of Solids, D-01187 Dresden, Germany

**Keywords:** cuprate, superconductor, STM, SJTM, superexchange

## Abstract

The elementary CuO_2_ plane sustaining cuprate high-temperature superconductivity occurs typically at the base of a periodic array of edge-sharing CuO_5_ pyramids. Virtual transitions of electrons between adjacent planar Cu and O atoms, occurring at a rate t/ℏ and across the charge-transfer energy gap E, generate “superexchange” spin–spin interactions of energy $J\approx 4t^{4}/E^{3}$ in an antiferromagnetic correlated-insulator state. However, hole doping this CuO_2_ plane converts this into a very-high-temperature superconducting state whose electron pairing is exceptional. A leading proposal for the mechanism of this intense electron pairing is that, while hole doping destroys magnetic order, it preserves pair-forming superexchange interactions governed by the charge-transfer energy scale E. To explore this hypothesis directly at atomic scale, we combine single-electron and electron-pair (Josephson) scanning tunneling microscopy to visualize the interplay of E and the electron-pair density *n_P_* in Bi_2_Sr_2_CaCu_2_O_8+x_. The responses of both E and *n_P_* to alterations in the distance *δ* between planar Cu and apical O atoms are then determined. These data reveal the empirical crux of strongly correlated superconductivity in CuO_2_, the response of the electron-pair condensate to varying the charge-transfer energy. Concurrence of predictions from strong-correlation theory for hole-doped charge-transfer insulators with these observations indicates that charge-transfer superexchange is the electron-pairing mechanism of superconductive Bi_2_Sr_2_CaCu_2_O_8+x_.

## Significance

Charge-transfer superexchange interactions between electrons on adjacent Cu sites have long been hypothesized to generate the intense spin-singlet electron-pair formation in cuprate superconductors. But this concept is unproven, partly because there existed no analogue isotope effect in which one could controllably vary the charge-transfer energy E(r) and measure the changes in the electron-pair condensate Ψ. Our concept is to visualize both E(r) and $n_{P}\left(r\right)={\left|\Psi \right|}^{2}$ directly at atomic scale and as a function of varying apical oxygen displacements δ(r) that occur in Bi_2_Sr_2_CaCu_2_O_8+x_. These data provide access to controllable variations in E(r) and resultant effects on $n_{P}\left(r\right)$, yielding $dn_{P}/dE\approx -\;0.81\pm 0.17\;eV^{-1}$. This compares with recent prediction $dn_{P}/dE\approx -0.9\;eV^{-1}$ for superexchange-mediated electron pairing in Bi_2_Sr_2_CaCu_2_O_8+x_, indicating that charge-transfer superexchange is the electron-pairing mechanism in hole-doped superconductor Bi_2_Sr_2_CaCu_2_O_8+x_.

## Concept of electron pairing from charge-transfer superexchange interactions

**Fig. 1.**
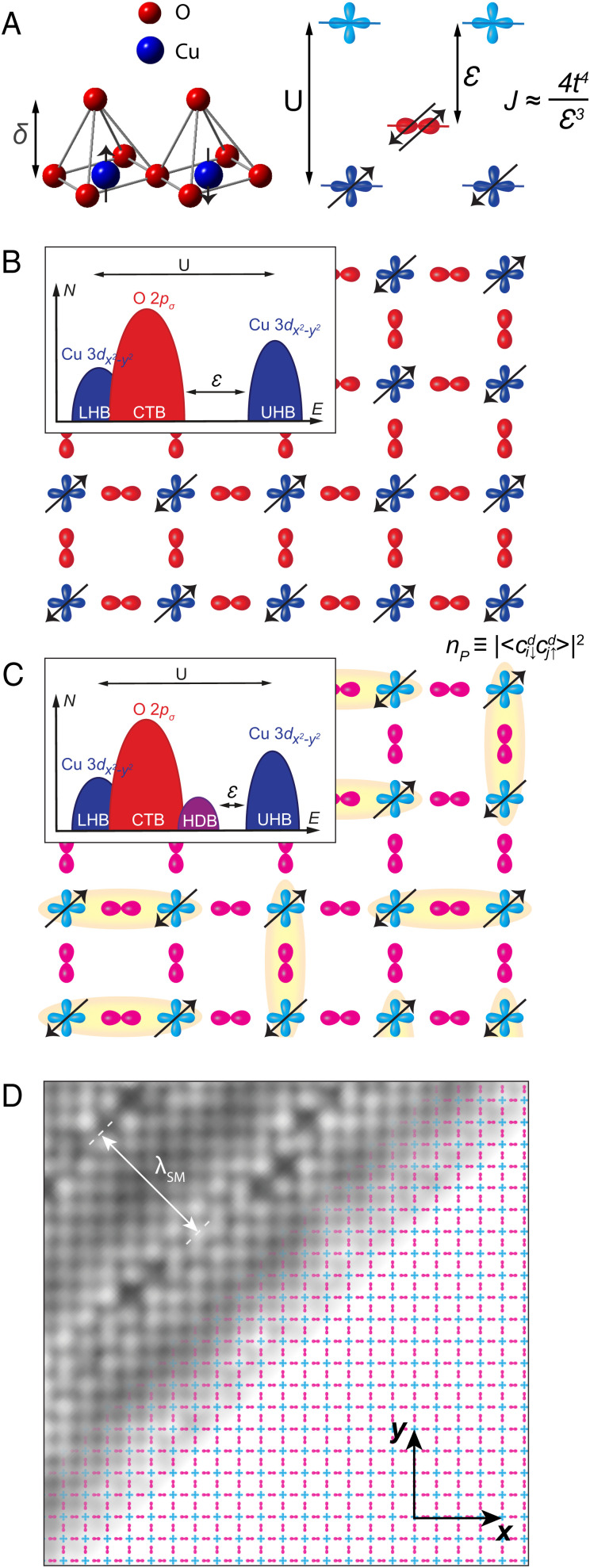
Superexchange magnetic interactions in transition-metal oxides. (*A*) Schematic representation of CuO_5_ pyramids whose bases comprise the CuO_2_ plane. The degeneracy of Cu $d_{x^{2}-y^{2}}$ orbitals (blue) is lifted by the Hubbard energy U, and the O $p_{\sigma }$ orbitals (red) are separated from the upper Cu $\;d_{x^{2}-y^{2}}\;$ band by the charge-transfer energy E (for holes). (*B*) Schematic of antiferromagnetic charge-transfer insulator state in undoped CuO_2_. Inset shows a schematic density of electronic states *N*(E) in this phase, with the Coulomb energy U and the charge-transfer energy E indicated. LHB, lower Hubbard band. UHB, upper Hubbard band. CTB, charge-transfer band. (*C*) Schematic of hole-doped CuO_2_, a two-dimensional correlated metallic state with intense antiferromagnetic spin–spin interactions. When superconductive, the electron-pair condensate $\Psi \equiv \langle c_{i↓}^{d}c_{j↑}^{d}\rangle$ is indicated schematically in yellow, and the related electron-pair density is $n_{P}\equiv {\left|\langle c_{i↓}^{d}c_{j↑}^{d}\rangle \;\right|}^{2}$. Inset shows a schematic *N*(E) in this phase that, although reorganized by the delocalized carriers, still retains a charge-transfer energy scale E. HDB, hole-doped band. (*D*) Schematic of CuO_2_ partially overlaid by a Bi_2_Sr_2_CaCu_2_O_8+x_ topographic image T(r) to exemplify how the crystal supermodulation modulates along the (1, 1) axis, with one period 0≤Φ≤2π requiring approximately 26 Å. The Cu to apical O distance δ is modulated at same wavevector but perpendicular to this plane.

1. The prospect that hole-doped CuO_2_ retains charge-transfer superexchange interactions between adjacent Cu spins has long motivated a hypothesis that spin-singlet electron-pair formation mediated by superexchange is the mechanism of high-temperature superconductivity. In transition-metal oxide insulators, superexchange (1) generates intense magnetic interactions between electrons that are localized at adjacent transition-metal atoms, typically generating robust antiferromagnetism. The superexchange interaction J occurs when the degeneracy of transition-metal 3d orbitals is lifted by the Coulomb energy U required for their double occupancy, so that intervening oxygen 2p energy levels are separated from the relevant transition-metal 3d level by the charge-transfer energy E. Within the framework of the three-band model, the interactions of two adjacent 3d electrons of spin $S_{i}\;$ are well approximated by a Heisenberg Hamiltonian $H=JS_{i}\cdot S_{j}$, with J the superexchange interaction produced by a multistage process of electronic exchange between spins on adjacent 3d orbitals via the nonmagnetic oxygen 2p orbitals. In the strong-coupling limit, $U/t\gg 1,\;J\approx 4t^{4}/E^{3}$, where the transition rate of electrons between 3d and 2p orbitals is given by *t/ℏ*. Specifically for CuO_2_-based materials, the planar Cu^2+^ ions are in the 3d^9^ configuration with a singly occupied $d_{x^{2}-y^{2}}$ orbital, while the planar O^2−^ ions have closed 2p^6^ shells whose in-plane $p_{\sigma }$ orbitals dominate. To doubly occupy any $d_{x^{2}-y^{2}}$ orbital requires an energy *U* so great that the *d* electrons become fully Mott localized in a charge-transfer insulator state, with the $p_{\sigma }\;$ energy level separated from the pertinent $d_{x^{2}-y^{2}}$ level by the CuO_2_ charge-transfer energy E (Fig. 1*A*). Under such circumstances, an electronic structure with t≈0.4 eV and E≈1 eV implies a superexchange energy J≈100 meV that should stabilize a robust spin-1/2, Q=(π,π) antiferromagnetic state (Fig. 1*B*). Just such a state is observed (2), confirming that charge-transfer superexchange is definitely the mechanism of the CuO_2_ antiferromagnetic state. However, when holes are doped into the CuO_2_ plane, they enter the $p_{\sigma }$ orbitals, both disrupting the antiferromagnetic order and delocalizing the electrons. This situation may be approximated using the three-band Hamiltonian based on a single Cu $d_{x^{2}-y^{2}}$ plus two O $p_{\sigma }$ orbitals per unit cell (3, 4):
  [1] $$H=\sum_{i\alpha j\beta ,\sigma }t_{ij}^{\alpha \beta }c_{i\sigma }^{†\alpha }c_{j\sigma }^{\beta }+\sum_{i\alpha ,\sigma }\varepsilon_{\alpha }n_{i\sigma }^{\alpha }+U\sum_{i}n_{i↑}^{d}n_{i↓}^{d}.$$
  Here, *i,j* enumerate planar CuO_2_ unit cells; *α,β* label any of the three orbitals; $t_{ij}^{\alpha \beta }$ are transition rates for electrons between orbitals *α,β* at sites *i,j*; $\varepsilon_{\alpha }$ are the orbital energies; and $n_{i↑}^{d},\;n_{i↓}^{d}$ are the $d_{x^{2}-y^{2}}$ orbital occupancies by spin state. Heuristically, such models describe a two-dimensional correlated metallic state with intense antiferromagnetic spin–spin interactions. If superconductivity occurs (Fig. 1*C*), it is signified by the appearance of a condensate of electron pairs $\Psi \equiv \langle c_{i↓}^{d}c_{j↑}^{d}\rangle ,\;$ a phenomenon that is now directly accessible to visualization using scanned Josephson tunneling microscopy (SJTM) (5–9).
2. Empirical study of charge-transfer superexchange as the mechanism of this superconductivity requires knowledge of the dependence of Ψ  on the charge-transfer energy E, but this has not been experimentally accessible. Certainly, E  and *J* have long been studied using optical reflectivity, Raman spectroscopy, tunneling spectroscopy, angle resolved photoemission, and resonant inelastic X-ray scattering (*SI Appendix*, section I). Typically, to access different E for these studies required changing between crystal families in the antiferromagnetic-insulator state. But this renders impossible the required comparison between E and Ψ measured simultaneously in the same superconductive state. Instead, the maximum superconducting critical temperature *T_C_* subsequent to hole doping is often proposed as a proxy for Ψ and then compared with the E derived from the parent insulator, for a range of different compounds. But varying the crystal family alters a wide variety of other material parameters besides E, and *T_C_* is anyway controlled by other influences, including dimensionality and superfluid phase stiffness (10). More fundamentally, advanced theoretical analysis has recently revealed that no one-to-one correspondence exists between the *T_C_* and Ψ in the CuO_2_ Hubbard model (11, 12). Hence, although greatly encouraging, studies comparing maximum superconductive *T_C_* with insulating E cannot be conclusive as to the electron-pairing mechanism. On the other hand, muon spin rotation studies do make clear that Ψ diminishes rapidly with increasing correlations upon approaching the charge-transfer insulator state (13). Ultimately, to identify the essential physics subtending this electron pairing, a direct and systematic measurement of the dependence of the electron-pair condensate Ψ  on the charge-transfer energy E at the same hole density is required.
3. In this context, dynamical mean-field theory analysis of the CuO_2_ Hubbard model has recently yielded quantitative predictions of how Ψ  is controlled by E. Moreover, theory also indicates that this interplay may be adjusted by altering the distance δ between each Cu atom and the apical O atom of its CuO_5_ pyramid (14–17). This is because varying δ should alter the Coulomb potential at the planar Cu and O atoms, modifying E and thereby controlling Ψ in a predictable manner (15–17), a scenario that has been advocated since the discovery of cuprate superconductivity (18–21). These realistically parameterized, quantitative predictions represent an exciting new opportunity: measurement of the dependences of Ψ on E at the Cu atom beneath each displaced apical oxygen atom, potentially yielding quantitative knowledge of dΨ/dE as a direct test for a charge-transfer superexchange electron-pairing mechanism (15–17). For experimentalists, the challenge is thus to measure the relationship between Ψ and E directly and simultaneously at the superconducting CuO_2_ plane. If available, such data could play a role analogous to the isotope effect in conventional superconductors (22), by identifying empirically for cuprates the specific electron–electron interaction that controls electron-pair formation.

## Techniques for visualization of charge-transfer energy and electron pair density

**Fig. 2.**
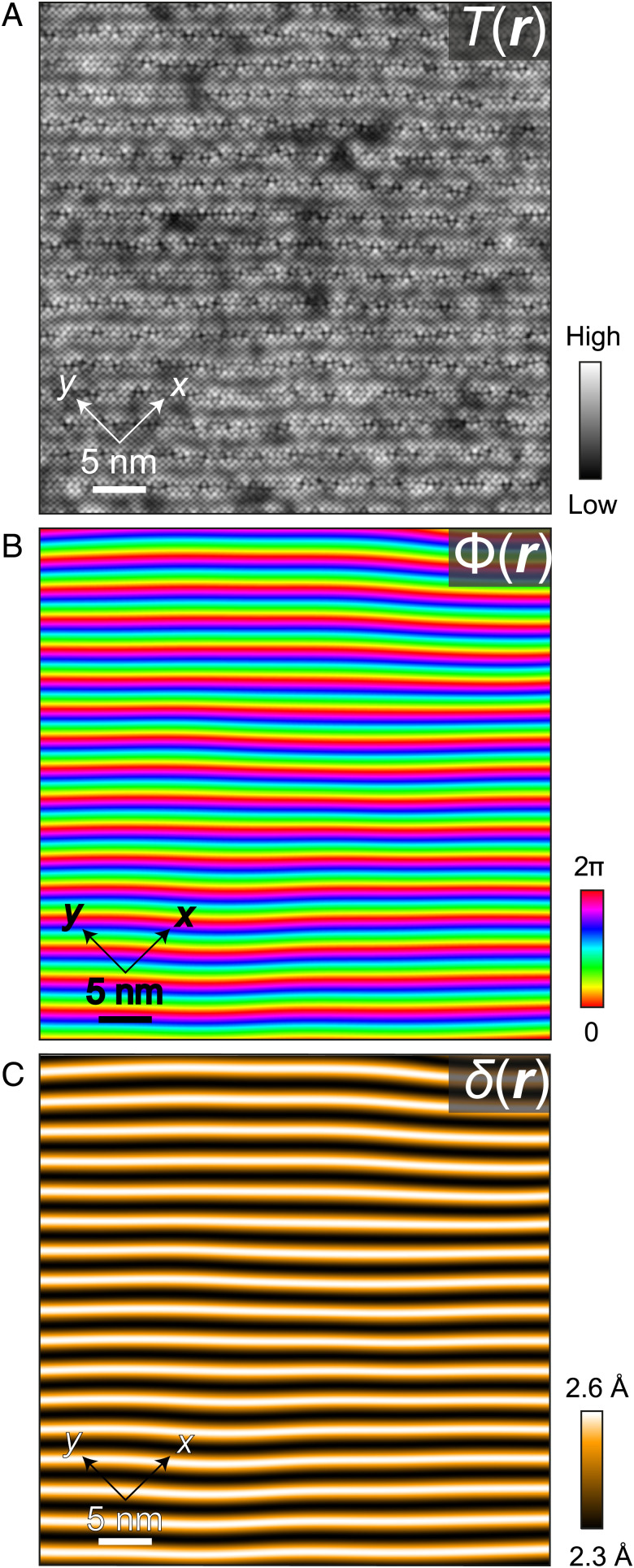
Imaging supermodulation phase Φ(r) and apical oxygen distance δ(r). (*A*) Exemplary Bi_2_Sr_2_CaCu_2_O_8+x_ topograph T(r) at the BiO termination layer. The planar Cu-O axes are at 45° to the supermodulation, as shown. The supermodulation runs from top to bottom with wavevector $Q_{S}\approx \left(0.15,0.15\right)2\pi /a_{0}$, obviously with relatively short correlation length. (*B*) From *A*, the supermodulation phase Φ(r) is derived (*SI Appendix*, section II). (*C*) From *B*, the apical distance δ(r) is derived from X-ray refinement data for the Bi_2_Sr_2_CaCu_2_O_8+x_ crystal structure (*SI Appendix*, section II).

4. To explore this prospect, one must measure Ψ and E as a function of separation *δ* above each planar Cu atom. But Ψ is, in general, a complex-valued field and thus not a physical observable, meaning that experimentalists must study ${\left|\Psi \right|}^{2}\equiv n_{P}$, the electron-pair density. Moreover, the pseudogap masks the true electron-pairing energy gap so that single-particle tunneling spectroscopy cannot be used to image the superconductive order parameter in lightly hole-doped cuprates. Our strategy therefore combines techniques in atomic-resolution imaging with a fortuitous property of the canonical cuprate Bi_2_Sr_2_CaCu_2_O_8+x_. First, a mismatch between preferred bond lengths of the rock-salt and perovskite layers in Bi_2_Sr_2_CaCu_2_O_8+x_ generates a λ ∼ 26Å periodic modulation of unit-cell dimensions (Fig. 1*D*), along the crystal *a* axis or equivalently the (1,1) axis of the CuO_2_ plane (23). Providentially, this crystal supermodulation generates periodic variations in *δ* by up to 12% in the single-electron excitation spectrum (24) and in the electron-pair (Josephson) current (7). However, the influence of the supermodulation on E and $n_{P}$ was unknown. Crucially for our objectives, the value of δ at every location ***r*** can be evaluated by atomic-resolution imaging of the supermodulation in topographic images T(r) measured at the crystal’s BiO termination layer (Figs. 1*D* and 2*A*) and then by using X-ray crystallography to relate T(r) to the spatial pattern of apical displacements δ(r) just underneath (*SI Appendix*, section II). Second, by measuring differential tunnel conductance dI/dV(r,V)≡g(r,V) as a function of location ***r*** and tip-sample voltage V, the density of electronic states N(r,E) ∝ g(r,V=E/e) can be visualized for the high energy range governed by Eq. 1. In principle, this allows energy scales, such as E(r) in the spectrum of Bi_2_Sr_2_CaCu_2_O_8+x_, to be determined versus location ***r***. Third, using superconducting scanning tunneling microscope (STM) tips (Bi_2_Sr_2_CaCu_2_O_8+x_ nanoflake tips (7)) to image the Josephson critical current $I_{\text{J}}$ for electron-pair tunneling versus location ***r*** allows direct visualization of sample’s electron-pair density (7–9) $n_{P}(r)\equiv {\left|\Psi \right|}^{2}\;\propto \;{\left(I_{\text{J}}\left(r\right)R_{\text{N}}(r)\right)}^{2}$, where $R_{\text{N}}$ is the tip-sample normal state junction resistance. Thus, our concept is to visualize both E(r) and $n_{P}(r)$ directly at atomic scale, as a function of the apical oxygen displacements δ(r) that are produced by the crystal supermodulation in Bi_2_Sr_2_CaCu_2_O_8+x_.
5. In practice, single crystals of Bi_2_Sr_2_CaCu_2_O_8+x_ with hole-density p≈0.17 are cleaved in cryogenic ultrahigh vacuum in a dilution refrigerator-based spectroscopic imaging STM (SISTM) to reveal the BiO termination layer (Fig. 2*A*). The CuO_2_ plane is ∼ 5Å beneath the BiO surface and separated from it by the SrO layer containing the apical oxygen atom of each CuO_5_ pyramid (Fig. 1*A*). A surface corrugation T(r)=A(r)cosΦ(r), where $\Phi \left(r\right)=Q_{S}.r+\theta \left(r\right)$, occurs at the bulk supermodulation wavevector $Q_{S}≅\left(0.15,0.15\right)2\pi /a_{0}$, where θ(r) describes effects of disorder (Fig. 2*A*). The supermodulation phase Φ(r)  is then imaged by analyzing T(q), the Fourier transform of T(r), with typical results shown in Fig. 2*B* (*SI Appendix*, section II). X-ray scattering studies of the Bi_2_Sr_2_CaCu_2_O_8+x_ crystal supermodulation demonstrate that the distance to apical oxygen atom *δ* is minimal at Φ=0 and maximal at Φ=π, because the displacement amplitude of the *c* axis supermodulation is greater in the CuO_2_ layer than in the adjacent SrO layer. Thus, δ(r) is determined from the measured Φ(r) based on X-ray refinement as δ(Φ)≈2.44−0.14cos(Φ) Å (*SI Appendix*, section II). For example, the apical displacement imaging results δ(r) from Fig. 2 *A* and *B* are shown in Fig. 2*C*. This same Φ(r):δ(r) procedure is used throughout our study.

## Coterminous visualization of charge-transfer energy and electron pair density

**Fig. 3.**
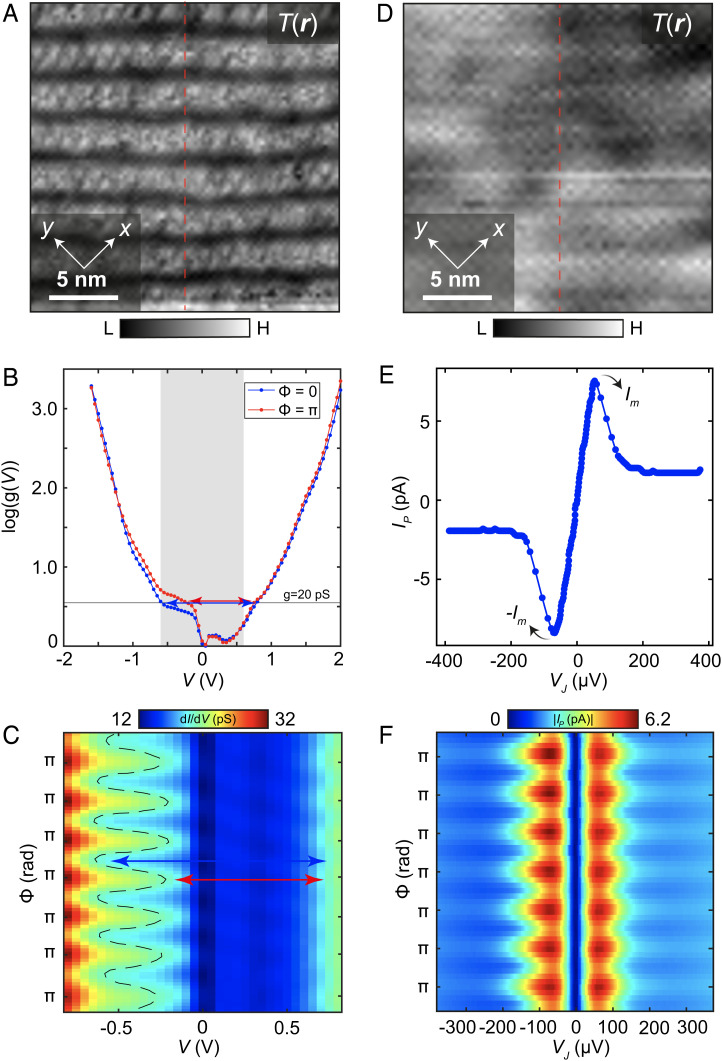
Visualizing charge-transfer energy E and electron-pair density $n_{P}$. (*A*) Topographic image of BiO termination layer at *T* = 4.2 K, using a nonsuperconducting W-tip. Trajectory of dashed red line corresponds to the data in *C*. (*B*) g(V) spectra of single-electron tunnelling measured at high-voltage and high tunnel junction resistance $R_{N}\approx 85\;\text{GΩ}\;$ in the FOV of *A* averaged at supermodulation phases Φ=0 and Φ=π. Use of logarithmic scale log(g(V)) reveals exponential growth of density of states away from gap edges (28). The estimated value of E is derived as the minimum energy separation between the bands at constant g=20  pS, as shown by double-head arrows. The value of E is shown to change by ≈0.3 eV from Φ=0 to Φ=π (*SI Appendix*, section III). (*C*) Measured g(V) along the dashed line in *A*. The energy difference E between the lower and upper gap edge is very clearly modulating, with typical examples of E(Φ=0) and E(Φ=π) indicated by blue and red double-headed arrows, respectively. (*D*) Topographic image of BiO termination layer at *T* = 2.1 K, using a superconducting tip. Trajectory of dashed red line corresponds to the data in *F*. (*E*) Typical $I_{P}(V_{J})$ spectrum of electron-pair tunnelling measured at low voltage and $R_{N}\approx 21\;\text{MΩ}$ in the FOV of *D*. (*F*) Measured $\left|I_{P}\left(V_{J}\right)\right|$ along the dashed linecut in *D*. The maxima of the electron-pair current are very clearly modulating at the same wavevector as in *C*. Though not a direct measure of $n_{p}(r)$, this gives the most direct empirical indication that supermodulations are occurring in the pair density. The minima(maxima) in $\left|I_{P}\left(V_{J}\right)\right|$ occur at Φ=m2π(Φ=(2m+1)π), where *m* is an integer. We note that it is the maxima(minima) in the pseudogap energy as measured by single-particle tunnelling that occur at the equivalent phases of the supermodulation (24), as might be expected from the relationship between pseudogap and condensed pair density in the cuprate phase diagram. For clarity, *C* and *F* have been Fourier filtered at the crystal supermodulation wavevector.

**Fig. 4.**
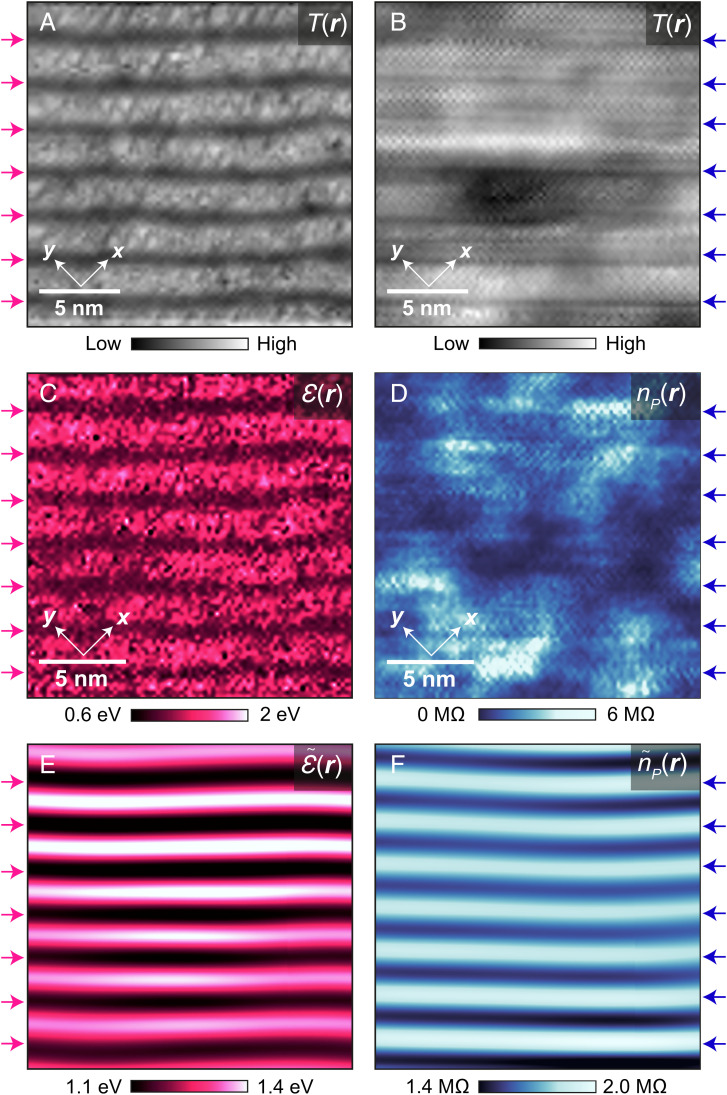
Atomic-scale visualization of E(r) and $n_{P}(r)$ versus δ(r). (*A*) Topographic image T(r) simultaneous with high-voltage g(r,V) measured at $R_{N}\approx 85\;\text{GΩ}$, yielding *C*. The pink arrowheads are at supermodulation Φ=π, as determined using the procedures described in *SI Appendix*, section II. (*B*) Topographic image T(r) simultaneous with low-voltage $I_{P}\left(r,V_{J}\right)$ and $R_{N}\left(r\right)$ maps, yielding *D*. The blue arrowheads are at Φ=π, as determined using the procedures described in *SI Appendix*, section II. The topographic image has atomic resolution, allowing the BiO layer to be discerned clearly, although it is somewhat different from *A*, due to use of a Bi_2_Sr_2_CaCu_2_O_8+x_ nanoflake superconductive tip (7) (*SI Appendix*, section IV). (*C*) Measured E(r) in the FOV of *A*. The mean value is E=1.195 eV, which is in very good agreement with E(r) for Bi_2_Sr_2_CaCu_2_O_8+x_ derived independently from other techniques (*SI Appendix*, section III). The pink arrowheads are at Φ=π of the supermodulation. (*D*) Measured $n_{P}(r)\;$ in the FOV of (*B*) (*SI Appendix*, section IV). The blue arrowheads are at Φ=π. (*E*) Fourier filtered $\tilde{E}\left(r\right)$ at supermodulation wavevectors $\pm Q_{S}$ in the FOV of *A* and *C*. The pink arrowheads are at Φ=π. (*F*) Fourier filtered ${\tilde{n}}_{P}(r)\;$ at supermodulation wavevectors $\pm Q_{S}$ in the FOV of *B* and *D*. The blue arrowheads are at Φ=π.

**Fig. 5.**
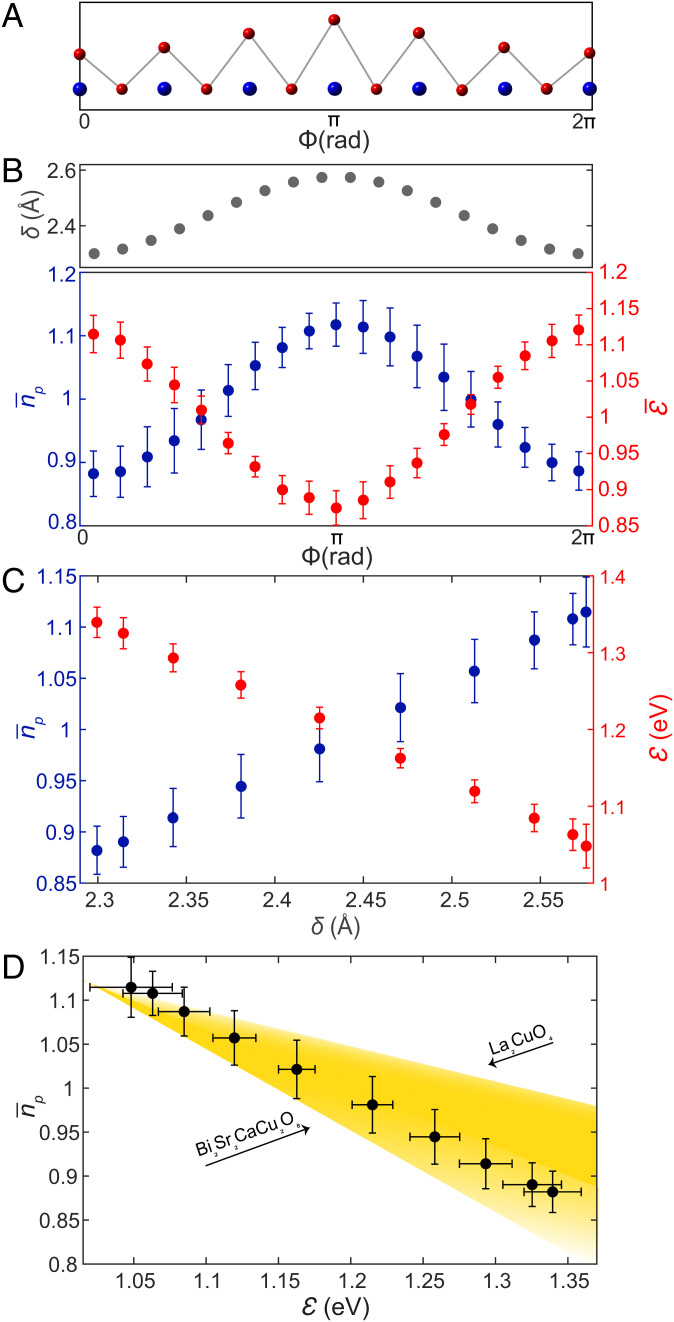
Evolution of cuprate electron-pair density $n_{P}$ with charge-transfer gap E. (*A*) Schematic of planar Cu to apical O distance modulations δ(r) in Bi_2_Sr_2_CaCu_2_O_8+x_ shown versus supermodulation phase Φ. (*B*) Gray dots: δ(Φ) showing the displacement of the apical oxygen atom within the CuO_5_ pyramid versus supermodulation phase Φ (23). Red dots: measured $\bar{E}\left(\Phi \right)$ showing the typical value for the Cu-O charge-transfer energy E for each value of the supermodulation phase Φ normalized to the mean value of E. These data are from the same FOV as Fig. 4 *A*, *C*, and *E*. Blue dots: measured ${\bar{n}}_{P}\left(\Phi \right)$ showing the measured value of electron-pair density versus supermodulation phase Φ. These data are from a larger FOV comprising 13 supermodulation periods, which contains the FOV from Fig. 4 *B*, *D*, and *F*. (*C*) Measured dependence of Cu-O charge-transfer energy E and electron-pair density $n_{P}$ on the displacement δ  of the apical O atoms from the planar Cu atoms. (*D*) Measured relationship of electron-pair density ${\bar{n}}_{P}$ to the Cu-O charge-transfer energy E in the CuO_2_ plane of Bi_2_Sr_2_CaCu_2_O_8+x_. The yellow shaded region shows the range of predicted slopes for $d{\bar{n}}_{P}/dE\equiv -\alpha \;{\text{eV}}^{-1}$, as $0.3\;≲\;\alpha \;≲\;1.0\;{\text{eV}}^{-1}$. These are derived from dynamical mean-field theory calculations for various materials with the limits reported for La_2_CuO_4_ and Bi_2_Sr_2_CaCu_2_O_8+x_, as indicated by black arrows. Error bars for *B*, *C*, and *D* are obtained from the standard deviation of the phase-averaged values.

6. In search of associated modulations in E(r), Fig. 3*A* shows a typical topographic image of the BiO termination layer, while Fig. 3*B* shows two high-voltage, single-electron g(V) spectra measured using junction resistance $R_{\text{N}}\approx 85\;\text{GΩ}$ in the same field of view. Such enormous junction resistances (or large tip-sample distances) preclude effects on g(V) of the tip-sample electric field. Hence, by visualizing g(r,V) in the −1.6 V≤V≤2 V range at these junction resistances, one can determine empirically whether E(r) modulations exist. For example, Fig. 3*B* shows representative g(r,V) spectra plotted on a logarithmic scale. We use the standard approach to estimate E as being the minimum energy difference between upper and lower bands (25) at a constant conductance G≈20 pS, as shown by double-headed arrows. This value of G implies no overlap in the measurements of E with the range of voltages |V|>0.9 V, where oxygen dopant atoms or vacancies cause significant disorder as indicated in *SI Appendix*, Fig. 4. Thus, the minimum energy separation between the top of the lower band and bottom of the upper band is indicated by the horizontal double-headed arrows (*SI Appendix*, section III). The blue arrows represent E(Φ=0) and the red arrows E(Φ=π). This is consistent with the well-established (26–28) value of charge-transfer energy E≈ 1.2 eV in Bi_2_Sr_2_CaCu_2_O_8_ (approximated by gray shaded region in Fig. 3*B*). Finally, plotting g(r,V) in Fig. 3*C*, along the trajectory shown by the dashed line Fig. 3*A*, reveals directly that E(r) modulates strongly at the supermodulation wavevector, with E(Φ=0)≈1.35eV (blue arrows) and E(Φ=π)≈0.95eV (red arrows), as indicated.
7. Correspondingly, to search for modulations in $n_{P}(r)$, Fig. 3*D* shows a typical topographic image of the BiO termination layer using a tip terminating in a Bi_2_Sr_2_CaCu_2_O_8+x_ nanoparticle (7). The junction resistance used here is $R_{\text{N}}\approx 21\;\text{MΩ}$; this is almost 5,000 times lower than that used for the E(r) studies, as are the typical electron-pair tunneling voltages $V_{\text{J}}$. Fig. 3*E* shows a typical $I_{P}(V_{\text{J}})$ spectrum measured in this field of view, with the tip-sample Josephson junction exhibiting a phase-diffusive steady state at voltage $V_{\text{J}},$ with electron-pair current $I_{P}\left(V_{\text{J}}\right)=\frac{1}{2}I_{\text{J}}^{2}ZV_{\text{J}}/\left(V_{\text{J}}^{2}+V_{\text{c}}^{2}\right)$, where *Z* is the high-frequency junction impedance and $V_{\text{C}}$ is the voltage for maximum $I_{P}\left(V_{\text{J}}\right)$. Then, because the maxima in $I_{P}\left(V_{\text{J}}\right)$ occur at $I_{\text{m}}\;\propto \;I_{\text{J}}^{2}$, atomic-scale visualization of an electron-pair density is achieved (7–9) as $n_{P}\left(r\right)\;\propto \;I_{\text{m}}(r)R_{N}^{2}\left(r\right)$ or equivalently $n_{\text{P}}\left(r\right)\;\propto \;g_{0}(r)R_{N}^{2}\left(r\right)$ (*SI Appendix*, section IV). In this study, we use the protocol $n_{\text{P}}\left(r\right)\;\propto \;g_{0}(r)R_{N}^{2}\left(r\right)$ to produce all key quantitative results as presented in Figs. 4 and 5. However, one can visualize empirically whether $n_{P}\left(r\right)$ modulations exist, by measuring $I_{P}\left(r,V_{\text{J}}\right)$ along the trajectory of the supermodulation (dashed line Fig. 3*D*). The result, as shown in Fig. 3*F*, clearly demonstrates how $\left|I_{\text{m}}\right|$ also modulates strongly at wavevector $Q_{S}$ (7).
8. Together, these data reveal that both the band-separation energy E(r) and the condensate electron-pair density$\;n_{P}\left(r\right)$ are modulated periodically by the crystal supermodulation of Bi_2_Sr_2_CaCu_2_O_8+x_. To quantify and relate these phenomena, we consider two exemplary fields of view whose T(r) are shown Fig. 4 *A* and *B*. Both T(r) images are evaluated to determine their separate Φ(r), with the ends of the Φ=π contours indicated by the arrowheads in each. A high-voltage, single-electron tunnelling g(r,V) map is measured at $R_{N}\approx 85\;\text{GΩ}$ and T=4.2 K in the field of view (FOV) of Fig. 4*A*, while a low-voltage, electron-pair tunnelling $I_{P}\left(V_{\text{J}}\right)$ map at $R_{N}\approx 21\;\text{MΩ}$ and T=2 K is measured in that of Fig. 4*B*. To visualize E(r), we estimate E to be the minimum energy difference between upper and lower bands (25) at a constant conductance G≈20 pS. The resulting E(r) shown in Fig. 4*C* is correctly representative and appears little different if we estimate E(r) anywhere in the range 20pS≤G≤80 pS (*SI Appendix*, section III). Concomitantly, to visualize $n_{P}(r)$, we measure $g_{0}\left(r\right)$ and multiply by the measured $R_{N}^{2}\left(r\right)$ modulations from the same FOV as Fig. 4*B*. The normal-state junction resistance $R_{N}(r)$ is obtained by self-normalizing two sets of dI/dV(r) spectra, one for $V_{\max}<\text{Δ}/\text{e}$ and the other for $V_{\max}>\text{Δ}/\text{e}$, measured in precisely the same FOV (*SI Appendix*, section IV). Thus, Fig. 4*D* shows measured $n_{P}(r)$ in the FOV of Fig. 4*B*. Finally, when Fig. 4*C* is Fourier filtered at $Q_{S}$, it reveals the first-harmonic modulations in $\tilde{E}(r)$, as presented in Fig. 4*E*, while identical filtering of Fig. 4*D* at $Q_{S}$ yields the first-harmonic modulations in ${\tilde{n}}_{P}\left(r\right)$, as seen in Fig. 4*F*. Thus, visualization of the crystal supermodulation effect on both E(r) and $n_{P}\left(r\right)$, simultaneously with their Φ(r), is now possible in Bi_2_Sr_2_CaCu_2_O_8+x_.

## Synthesis

9. So how does supermodulation displacement of the apical oxygen atom δ(r) (and to a lesser extent that of other atoms) alter the charge-transfer energy E(r) and the electron-pair density $n_{P}\left(r\right)$ at each planar Cu atom (15–21) in Bi_2_Sr_2_CaCu_2_O_8+x_? To synthesize data as in Fig. 4, we first plot apical distance alterations versus phase δ(Φ) for Bi_2_Sr_2_CaCu_2_O_8+x_, as shown by gray dots in Fig. 5*B*. We then process E(r) retaining only wavevectors close to $\pm Q_{S}$. Then, by corresponding simultaneous Φ(r):E(r) measurements (e.g., Fig. 4 *A* and *C*), we determine $\bar{E}\left(\Phi \right)$, whose value is normalized to the mean measured value and shown as red dots in Fig. 5*B*; this is found to be a very repeatable characteristic of Bi_2_Sr_2_CaCu_2_O_8+x_. Similarly, by corresponding simultaneous $\Phi \left(r\right):n_{P}\left(r\right)\;\text{measurements}$ (e.g., Fig. 4 *B* and *D*), we determine ${\bar{n}}_{P}\left(\Phi \right)$, which is normalized to the mean value of measured $n_{P}(r)$. This is shown by blue dots in Fig. 5*B*; this is another repeatable characteristic (*SI Appendix*, section VI). To maximize the precision of both the Fourier filtering and lock-in methods, we perform this analysis in an FOV that includes as many periods of the supermodulation as possible (for E(r), 7 periods, and for $n_{P}\left(r\right)$, 13 periods). The microscopic relationship of E to δ can then be determined by eliminating common variable Φ from Fig. 5*B*. The result, shown in Fig. 5*C*, provides a direct measurement of this long-sought characteristic (15–20) of cuprate electronic structure: dE/dδ≈−1.04±0.12 eV/Å and $d{\bar{n}}_{P}/d\delta \approx \;0.85\pm 0.22\;{Å}^{-1}$ for Bi_2_Sr_2_CaCu_2_O_8+x_. More fundamentally, the atomic-scale relationship between the normalized electron-pair density ${\bar{n}}_{P}$ and the charge-transfer energy E is derived by eliminating the common variable Φ. The result, as shown in Fig. 5*D*, demonstrates that $d{\bar{n}}_{P}/dE\approx -0.81\pm 0.17\;{\text{eV}}^{-1}$ or equivalently that $d|\bar{\left\langle c_{↑}c_{↓}\right\rangle }|/dE\approx -0.40\pm 0.09\;{\text{eV}}^{-1}$ over a wide range of charge-transfer energy scales in Bi_2_Sr_2_CaCu_2_O_8+x_.
10. Although the original predictions (15, 16) for dE/dδ were for La_2_CuO_4_, they are still in reasonable agreement with our observations for Bi_2_Sr_2_CaCuO_8+x_, as shown in Fig. 5*C*. Theoretical predictions for the direct effect on the cuprate electron-pair condensate of altering the charge-transfer E yield (*SI Appendix*, section VII) $d|\bar{\langle c_{↑}c_{↓}\rangle }|/dE\approx -\alpha /2\;{\text{eV}}^{-1}$ or equivalently $d{\bar{n}}_{P}/dE\approx -\alpha \;{\text{eV}}^{-1}$, with a range 0.3 ≲ α ≲ 1.0, depending on the material-specific parameters (11, 15–17). The precise parameters used in these calculations for a variety of different materials are given in ref. (15). Fig. 5*D* indicates the anticipated range of α for different materials using a yellow shaded triangle. For Bi_2_Sr_2_CaCu_2_O_8_ specifically (11), the three-band CuO_2_ Hubbard model prediction for a superexchange electron-pairing mechanism is that $d|\bar{\left\langle c_{↑}c_{↓}\right\rangle }|/dE\approx -0.46\pm 0.05\;{\text{eV}}^{-1}$ or equivalently that $\;\alpha \approx 0.93\pm 0.1\;{\text{eV}}^{-1}$. The agreement with experimental observations reported in Fig. 5*D* is self-evident.
11. For decades, the electron-pairing mechanism of cuprate high-temperature superconductivity has been hypothesized (29–36) as due to electron–electron interactions mediated by superexchange but with the electron-pair condensate Ψ subject to the strong no-double-occupancy constraints on the Cu $d_{x^{2}-y^{2}}$ orbitals (37, 38) (Fig. 1*C*). When such interactions and constraints were studied using mean-field Gutzwiller projection (37), by slave-boson techniques (38,39), or by Monte Carlo numerical techniques (36, 37), the phase diagram and many other key characteristics that emerged were congruent with observations (38, 39). Contemporary theoretical studies, using a wide variety of advanced theoretical and numerical techniques (39–44), also predict with growing confidence that it is the superexchange interaction that creates electron pairing in the three-band CuO_2_ Hubbard model. However, direct experimental tests of the relationship between the cuprate electron-pair condensate and the charge-transfer energy of this model were nonexistent. Here, by visualizing the electron-pair density $n_{P}(r)$ using SJTM (e.g., Fig. 4 *D* and *F*), and the charge-transfer energy E(r)  using high-voltage SISTM (e.g., Fig. 4 *C* and *E*), we find empirically that both modulate together at the Bi_2_Sr_2_CaCu_2_O_8+x_ crystal supermodulation wavevector ***Q_S_*** (Figs. 2 *B* and *C* and 5*B*). This joint $E(r):\;n_{\text{P}}(r)$ modulation is observed comprehensively throughout these studies of Bi_2_Sr_2_CaCu_2_O_8_, with its existence being independent of exactly which atomic displacements occur within the crystal supermodulation. The consequent demonstration that $d|\langle c_{↑}c_{↓}\rangle |/dE<0$ (Fig. 5*D*) is a direct visualization of an effect long anticipated in the theory of superexchange-mediated electron pairing in cuprates (3, 4, 14–17, 29–39) and from experiments based on muon spin rotation (13). More specifically, recent numerical studies of the three-band CuO_2_ Hubbard model (11), within which charge-transfer superexchange is demonstrably the cause of electron pairing (40–45), yield quantitative agreement between predicted $d|\bar{\langle c_{↑}c_{↓}\rangle }|/dE\approx -0.46\pm 0.05\;{\text{eV}}^{-1}$ and our experimental determination that $d{\bar{n}}_{\text{P}}/dE\approx -0.81\pm 0.17\;{\text{eV}}^{-1}$ for Bi_2_Sr_2_CaCu_2_O_8+x._ Taken at face value, the data in Fig. 5 thus indicate that charge-transfer superexchange is key to the electron-pairing mechanism of the hole-doped cuprate superconductor Bi_2_Sr_2_CaCu_2_O_8+x_.

## Supplementary Material

Supplementary File

## Data Availability

All data are included in the manuscript and/or *SI Appendix*.

## References

[1] P. W. Anderson, Antiferromagnetism. Theory of superexchange interaction. Phys. Rev. 79, 350 (1950).

[2] R. Coldea , Spin waves and electronic interactions in La_2_CuO_4_. Phys. Rev. Lett. 86, 5377–5380 (2001). 11384502 10.1103/PhysRevLett.86.5377

[3] V. J. Emery, Theory of high-*T_c_* superconductivity in oxides. Phys. Rev. Lett. 58, 2794–2797 (1987). 10034851 10.1103/PhysRevLett.58.2794

[4] P. B. Littlewood, C. M. Varma, E. Abrahams, Pairing instabilities of the extended Hubbard model for Cu-O-based superconductors. Phys. Rev. Lett. 63, 2602–2605 (1989). 10040937 10.1103/PhysRevLett.63.2602

[5] J. Šmakov, I. Martin, A. V. Balatsky, Josephson scanning tunneling microscopy. Phys. Rev. B Condens. Matter Mater. Phys. 64, 212506 (2001).

[6] M. Graham, D. K. Morr, Josephson scanning tunneling spectroscopy in $d_{x^{2}-y^{2}}$. Phys. Rev. Lett. 123, 017001 (2019). 31386405 10.1103/PhysRevLett.123.017001

[7] M. H. Hamidian , Detection of a Cooper-pair density wave in Bi_2_Sr_2_CaCu_2_O_8+x_. Nature 532, 343–347 (2016). 27074504 10.1038/nature17411

[8] D. Cho, K. M. Bastiaans, D. Chatzopoulos, G. D. Gu, M. P. Allan, A strongly inhomogeneous superfluid in an iron-based superconductor. Nature 571, 541–545 (2019). 31341304 10.1038/s41586-019-1408-8

[9] X. Liu, Y.-X. Chong, R. Sharma, J. C. Davis, Discovery of a Cooper-pair density wave state in a transition-metal dichalcogenide. Science 372, 1447 (2021).

[10] V. J. Emery, S. A. Kivelson, Importance of phase fluctuations in superconductors with small superfluid density. Nature 374, 434–437 (1995).

[11] N. Kowalski, S. S. Dash, P. Sémon, D. Sénéchal, A.-M. S. Tremblay, Oxygen hole content, charge-transfer gap, covalency, and cuprate superconductivity. Proc. Natl. Acad. Sci. U.S.A. 118, e2106476118 (2021). 34593641 10.1073/pnas.2106476118 PMC8501840

[12] P. Mai, G. Balduzzi, S. Johnston, T. A. Maier, Pairing correlations in the cuprates: A numerical study of the three-band Hubbard model. Phys. Rev. B 103, 144514 (2021).

[13] Y. J. Uemura , Universal correlations between T_c_ and $\frac{n_{s}}{m^{*}}$ (carrier density over effective mass) in high-Tc cuprate superconductors. Phys. Rev. Lett. 62, 2317–2320 (1989). 10039913 10.1103/PhysRevLett.62.2317

[14] C. Weber, K. Haule, G. Kotliar, Strength of correlations in electron- and hole-doped cuprates. Nat. Phys. 6, 574–578 (2010).

[15] C. Weber, C. Yee, K. Haule, G. Kotliar, Scaling of the transition temperature of hole-doped cuprate superconductors with the charge-transfer energy. Europhys. Lett. 100, 37001 (2012).

[16] C.-H. Yee, G. Kotliar, Tuning the charge-transfer energy in hole-doped cuprates. Phys. Rev. B Condens. Matter Mater. Phys. 89, 094517 (2014).

[17] S. Acharya , Metal-insulator transition in copper oxides induced by apex displacements. Phys. Rev. X 8, 021038 (2018).

[18] Y. Ohta, T. Tohyama, S. Maekawa, Electronic structure of insulating cuprates: Role of Madelung potential in the charge-transfer gap and superexchange interaction. Physica C 185-189, 1721–1722 (1991). 10.1103/PhysRevLett.66.1228 10044028

[19] L. F. Feiner, M. Grilli, C. Di Castro, Apical oxygen ions and the electronic structure of the high-T_c_ cuprates. Phys. Rev. B Condens. Matter 45, 10647–10669 (1992). 10000972 10.1103/physrevb.45.10647

[20] E. Pavarini, I. Dasgupta, T. Saha-Dasgupta, O. Jepsen, O. K. Andersen, Band-structure trend in hole-doped cuprates and correlation with T_c max_. Phys. Rev. Lett. 87, 047003 (2001). 11461638 10.1103/PhysRevLett.87.047003

[21] K. Foyevtsova, R. Valenti, P. J. Hirschfeld, Effect of dopant atoms on local superexchange in cuprate superconductors: A perturbative treatment. Phys. Rev. B Condens. Matter Mater. Phys. 79, 144424 (2009).

[22] C. A. Reynolds, B. Serin, W. H. Wright, L. B. Nesbitt, Superconductivity of isotopes of mercury. Phys. Rev. 78, 487 (1950).

[23] Y. Gao, P. Lee, P. Coppens, M. A. Subramania, A. W. Sleight, The incommensurate modulation of the 2212 bi-sr-ca-cu-o superconductor. Science 241, 954–956 (1988). 17731444 10.1126/science.241.4868.954

[24] J. A. Slezak , Imaging the impact on cuprate superconductivity of varying the interatomic distances within individual crystal unit cells. Proc. Natl. Acad. Sci. U.S.A. 105, 3203–3208 (2008). 18287001 10.1073/pnas.0706795105 PMC2265138

[25] P. Cai , Visualizing the evolution from the Mott insulator to a charge-ordered insulator in lightly doped cuprates. Nat. Phys. 12, 1047–1051 (2016).

[26] W. Ruan , Relationship between the parent charge transfer gap and maximum transition temperature in cuprates. Sci. Bull. (Beijing) 61, 1826–1832 (2016).

[27] S.-L. Yang , Revealing the Coulomb interaction strength in a cuprate superconductor. Phys. Rev. B 96, 245112 (2017).

[28] T. Itoh, K. Fueki, Y. Tanaka, H. Ihara, Optical conductivity spectra and electronic structure of Bi_2_Sr_2_(Y_1−x_Ca_x_)Cu_2_O_y_ system. J. Phys. Chem. Solids 60, 41–51 (1999).

[29] P. W. Anderson, The resonating valence bond state in La_2_CuO_4_ and superconductivity. Science 235, 1196–1198 (1987). 17818979 10.1126/science.235.4793.1196

[30] F. C. Zhang, T. M. Rice, Effective Hamiltonian for the superconducting Cu oxides. Phys. Rev. B Condens. Matter. 37, 3759–3761 (1988). 9944993 10.1103/physrevb.37.3759

[31] F. C. Zhang, C. Gros, T. M. Rice, H. Shiba, A renormalised Hamiltonian approach to a resonant valence bond wavefunction. Supercond. Sci. Technol. 1, 36–46 (1988).

[32] A. E. Ruckenstein, P. J. Hirschfeld, J. Appel, Mean-field theory of high-Tc superconductivity: The superexchange mechanism. Phys. Rev. B Condens. Matter. 36, 857–860 (1987). 9942134 10.1103/physrevb.36.857

[33] G. Kotliar, J. Liu, Superexchange mechanism and d-wave superconductivity. Phys. Rev. B Condens. Matter. 38, 5142–5145 (1988). 9946940 10.1103/physrevb.38.5142

[34] P. A. Lee, N. Nagaosa, T.-K. Ng, X.-G. Wen, SU(2) formulation of the t−J model: Application to underdoped cuprates. Phys. Rev. B 57, 6003–6021 (1998).

[35] R. T. Scalettar, D. J. Scalapino, R. L. Sugar, S. R. White, Antiferromagnetic, charge-transfer, and pairing correlations in the three-band Hubbard model. Phys. Rev. B Condens. Matter. 44, 770–781 (1991). 9999180 10.1103/physrevb.44.770

[36] M. Paramekanti, M. Randeria, N. Trivedi, Projected wave functions and high temperature superconductivity. Phys. Rev. Lett. 87, 217002 (2001). 11736370 10.1103/PhysRevLett.87.217002

[37] P. W. Anderson , The physics behind high-temperature superconducting cuprates: The ‘plain vanilla’ version of RVB. J. Phys. Condens. Matter 16, R755 (2004).

[38] P. A. Lee, N. Nagaosa, X.-G. Wen, Doping a Mott insulator: Physics of high-temperature superconductivity. Rev. Mod. Phys. 78, 17 (2006).

[39] D. Poilblanc, D. J. Scalapino, Calculation of Δ(k, ω) for a two-dimensional t − J cluster. Phys. Rev. B Condens. Matter Mater. Phys. 66, 052513 (2002).

[40] K. Haule, G. Kotliar, Strongly correlated superconductivity: A plaquette dynamical mean-field theory study. Phys. Rev. B 76, 104509 (2007).

[41] T. A. Maier, D. Poilblanc, D. J. Scalapino, Dynamics of the pairing interaction in the Hubbard and t − J models of high-temperature superconductors. Phys. Rev. Lett. 100, 237001 (2008). 18643535 10.1103/PhysRevLett.100.237001

[42] S. S. Kancharla , Anomalous superconductivity and its competition with antiferromagnetism in doped Mott insulators. Phys. Rev. B 77, 184516 (2008).

[43] E. Gull, A. J. Millis, Pairing glue in the two-dimensional Hubbard model. Phys. Rev. B 90, 041110(R) (2014).

[44] A. T. Rømer , Pairing in the two-dimensional Hubbard model from weak to strong coupling. Phys. Rev. Res. 2, 013108 (2020).

