# Supplementary material for: On the electron pairing mechanism of copper-oxide high temperature superconductivity
Source: Proc Natl Acad Sci U S A. 2022 Sep 6;119(37):e2207449119. doi: 10.1073/pnas.2207449119 (PMC9477408; doi:10.1073/pnas.2207449119)
Supplement: Supplementary File [file pnas.2207449119.sapp.pdf]

Supplementary Material for

On the Electron Pairing Mechanism of Copper-Oxide High Temperature Superconductivity

S.M. O'Mahony, Wangping Ren, Weijiong Chen, Yi Xue Chong, Xiaolong Liu,
H. Eisaki, S. Uchida, M.H. Hamidian, and J.C. Séamus Davis

(I) Charge Transfer Energy, Superexchange and Superconductivity

Many types of studies of $\mathcal{E}$ and its consequent J for cuprates have been carried out on bulk crystals. Optical reflectivity (*46,47*) revealed that the charge transfer energy $\mathcal{E}$ in the parent insulating state (Fig. 1b), for virtually all cuprates that become superconducting under hole-doping, is $1\ \mathrm{eV} < \mathcal{E} < 2\ \mathrm{eV}$. Raman scattering (*46,47,48*) identified the concomitant electron-pair excitations as a superexchange energy in the range $J{\sim}150$ meV. Single-electron tunneling spectroscopy (*27*) reveals that the charge transfer energy for $Bi_2Sr_2CaCu_2O_{8+x}$ in the insulating parent state is $\mathcal{E} \gtrsim 1$ eV, and a non-monotonic but diminishing trend of maximum T_C measured in the superconductor with increasing $\mathcal{E}$ measured in the corresponding insulator, for samples of several distinct material types. Advanced angle resolved photoemission (*49*) reports transitions to unoccupied states consistent with a Cu on-site Coulomb interaction strength U of 2.7 eV and that the charge-transfer energy of optimally doped $Bi_2Sr_2CaCu_2O_{8+x}$ is then $\mathcal{E} \approx$ 1.1 eV. Resonant inelastic X-ray scattering (*50,51*) reveals directly, from the spectrum of spin wave excitations of many cuprate materials, that $140\ \mathrm{meV} < J < 180\ \mathrm{meV}$. Most recently (*52*) X-ray absorption spectra at both the Cu-L edge and O-K edge were used to determine $\mathcal{E} \approx 1.3\ \mathrm{eV}$ in optimally superconducting $(Ca_xLa_{1-x})(Ba_{1.75-x}La_{0.25+x})Cu_3O_y$. Nevertheless, despite all these pioneering studies of $\mathcal{E}$ and J for cuprates, simultaneous measurements of effects on the cuprate electron-pair condensate amplitude $\langle c_i^\dagger c_j^\dagger \rangle$ of either $\mathcal{E}$ or J have never, to our knowledge, been reported.

(II) Supermodulation Phase $\boldsymbol{\Phi}(\boldsymbol{r})$ and Apical Displacement $\boldsymbol{\delta}(\boldsymbol{r})$ Estimation

The bulk crystal supermodulation in $Bi_2Sr_2CaCu_2O_{8+x}$ perturbs the atoms from their idealized crystal positions due to the mismatch between the bond lengths of the rock-salt layer and the perovskite layer of $Bi_2Sr_2CaCu_2O_{8+x}$. The modulation has a periodicity of ~ 26 Å along the a-axis of the crystal (*53*) but lacks long range coherence. The distance between all atoms in each $Bi_2Sr_2CaCu_2O_{8+x}$ unit cell modulates periodically, with the change in distance between the planar Cu atom and the apical O atom being largest in amplitude (Fig. S1) varying between 2.25 Å and 2.54 Å over one supermodulation period (Fig 5a). X-ray scattering data also reveals that the Bi-O distance is virtually constant over the supermodulation period (Fig. S1), enabling direct connection between the supermodulation signal in the topographic image $T(\boldsymbol{r})$ and the modulation of the planar Cu to apical O separation $\delta(\boldsymbol{r})$ (Fig. S1). Fig. S1 also shows the displacement of the BiO termination layer from the symmetry plane of the crystal, which

is equivalent to a topograph $T(\boldsymbol{r})$. This allows one to directly relate the supermodulation phase Φ obtained from $T(\boldsymbol{r})$ to the X-ray refinement data (*53*).

Effects of the supermodulation can be observed in topographic images $T(\boldsymbol{r})$ measured at the BiO crystal termination layer of $Bi_2Sr_2CaCu_2O_{8+x}$. Its signal as a component of the topographic image can be expressed as

$$T'(\boldsymbol{r}) = A_S(\boldsymbol{r})\cos\Phi(\boldsymbol{r}), \quad \text{(S1)}$$

$$\Phi(\boldsymbol{r}) = \boldsymbol{Q_S}\cdot\boldsymbol{r} + \theta(\boldsymbol{r}), \quad \text{(S2)}$$

where $\boldsymbol{Q_S}$ is the supermodulation vector and $\theta(\boldsymbol{r})$ is a spatially dependent phase disorder. To measure $\Phi(\boldsymbol{r})$ we process $T(\boldsymbol{r})$ retaining only the wavevector components near $\boldsymbol{q} = \pm\boldsymbol{Q_S}$. In practice, this is achieved by first Fourier filtering $T(\boldsymbol{r})$ as follows:

$$T'(\boldsymbol{r}) = \frac{1}{\sigma\sqrt{2\pi}}\int T(\boldsymbol{q})\left(e^{\frac{|\boldsymbol{q}-\boldsymbol{Q_S}|^2}{2\sigma^2}} + e^{\frac{|\boldsymbol{q}+\boldsymbol{Q_S}|^2}{2\sigma^2}}\right)\exp(-i\boldsymbol{q}\cdot\boldsymbol{r})\,\mathrm{d}\boldsymbol{q} \quad \text{(S3)}$$

where $T(\boldsymbol{q})$ is the Fourier transform of $T(\boldsymbol{r})$. We use $\sigma \approx \frac{2\pi}{20}$ nm^{-1} when filtering $\mathcal{E}(\boldsymbol{r})$ and $\sigma \approx \frac{2\pi}{40}$ nm^{-1} when filtering $n_P(\boldsymbol{r})$, to take account of the difference in tip sizes. Next, to visualize the phase disorder $\theta(\boldsymbol{r})$, we employ a two-dimensional lock-in method in which references $\alpha(\boldsymbol{r}) = \sin(\boldsymbol{Q_S}\cdot\boldsymbol{r})$ and $\beta(\boldsymbol{r}) = \cos(\boldsymbol{Q_S}\cdot\boldsymbol{r})$ are multiplied by $T'(\boldsymbol{r})$:

$$X(\boldsymbol{r}) \equiv T'(\boldsymbol{r})\alpha(\boldsymbol{r}), \quad (S4)$$

$$Y(\boldsymbol{r}) \equiv T'(\boldsymbol{r})\beta(\boldsymbol{r}). \quad (S5)$$

Low pass filtering these product images to remove the AC term, yields

$$\theta(\boldsymbol{r}) = \arctan\left(\frac{Y(\boldsymbol{r})}{X(\boldsymbol{r})}\right). \quad (S6)$$

Adding back the pure modulating $\boldsymbol{Q_S}\cdot\boldsymbol{r}$ term and defining the result modulo 2π, generates a map of the total supermodulation phase $\Phi(\boldsymbol{r}) = [\boldsymbol{Q_S}\cdot\boldsymbol{r} + \theta(\boldsymbol{r})] \bmod 2\pi$.

The relationship between the apical Cu-O displacement δ and phase Φ can then be obtained using X-ray refinement techniques (*53*) that report apical displacement varies between $\delta \sim 2.25$ Å at $\Phi = 0$ and $\delta \sim 2.54$ Å at $\Phi = \pi$ (Fig. S1) The first harmonic is reported as:

$$\delta(\Phi) = 2.44 - 0.14\cos(\Phi)\ \text{Å} \quad \text{(S7)}$$

This enables to make direct connection between the measured supermodulation phase $\Phi(\boldsymbol{r})$ obtained from the topographic image $T(\boldsymbol{r})$ and the Cu-O separation $\delta(\boldsymbol{r})$ within each crystal unit cell. Fig. S2 shows a typical example of the equivalent $T(\boldsymbol{r})$:$\Phi(\boldsymbol{r})$:$\delta(\boldsymbol{r})$ arising from this procedure when using a $Bi_2Sr_2CaCu_2O_{8+x}$ nanoflake tip for SJTM measurements of $n_P(\boldsymbol{r})$.

(III) Charge Transfer Energy Visualization $\mathcal{E}(\boldsymbol{r})$ by SISTM

To extract the spatially resolved charge transfer energy $\mathcal{E}(\boldsymbol{r})$, high voltage *(26,54,55,*) differential tunnel conductance $g(\boldsymbol{r},V)$ imaging of $Bi_2Sr_2CaCu_2O_{8+x}$ is used. Here, we visualize $g(\boldsymbol{r},V)$ in the range $-1.6\ \text{V} \le V \le 2\ \text{V}$, and at the extremely high junction resistance $R_N \approx 85$ GΩ ($V_S = 600$ mV: $I_S = 7$pA) necessary to suppress all tip-induced electric field effects. Fig. S3g then shows a typical example of a $g(\boldsymbol{r},V)$ series

measured at T=4.2K along a line transverse to the crystal supermodulation. The edges of the filled lower band and the empty upper band can be identified from the appearance of extremely rapid increase in the density of states. Here, as Fig S3g, we produce Fig. 3c from the main text showing the measured $g(\boldsymbol{r}, V)$ in which these $g(\boldsymbol{r}, V)$ variations at the periodicity as the supermodulation are clearly evident.

One must consider the possibility of a "setup effect" on these data. Most simply, the electron tunneling current from the STM tip to the sample is

$$I(\boldsymbol{r}, V) = Ce^{-\frac{T(r)}{T_0}} \int_0^{E=eV} [f(E,T)N(\boldsymbol{r}, E)]\big[(1 - f(E,T))N_{\mathrm{Tip}}(E)\big]dE \qquad \text{(S8)}$$

where $T(\boldsymbol{r})$ is the 'topograph', V the tip-sample bias voltage, N ($\boldsymbol{r}$, E) the sample's local-density-of-electronic-states, $N_{\mathrm{TIP}}(E)$ the tip density-of-electronic-states $f(E,T)$ is the Fermi function. Here $Ce^{-\frac{T(r)}{T_0}}$ contains all the effects of tip elevation, tip-sample work-functions and tunneling matrix element. Thus, at very low temperatures and with N_{TIP}(E) and $C(\boldsymbol{r})$ both constants

$$I_s = Ce^{-\frac{T(r)}{T_0}} \int_0^{eV_s} N(\boldsymbol{r}, E)dE \Rightarrow Ce^{-\frac{T(r)}{T_0}} = I_s / \int_0^{eV_s} N(\boldsymbol{r}, E)dE \qquad \text{(S9)}$$

where V_S and I_S are the (constant but arbitrary) 'set-up' bias voltage and current respectively. In practice, these two parameters fix the tip elevation $T(\boldsymbol{r})$ for each tip-sample junction. Equivalently, standard constant-current topographic imaging adjusts $T(\boldsymbol{r})$ as the tip scans over the sample surface to maintain a set point current, I_S, at a constant applied tip-sample bias V_S. In this case, the topographic image $T(\boldsymbol{r}, V_S)$ is

$$T(\boldsymbol{r}, V_S) = \mathrm{T}_0 \ln\left[\int_0^{E=eV_S} N(\boldsymbol{r}, E)dE\right] \qquad \text{(S10)}$$

up to a constant. The energy derivative of Eq. S8 at zero temperature yields the tip-sample differential conductance $dI/dV(\boldsymbol{r}, E = eV) \equiv g(\boldsymbol{r}, E)$ which, upon substitution of $Ce^{-\frac{T(r)}{T_0}}$ from Eq. S9 yields

$$g(\boldsymbol{r}, V) = eI_s N(\boldsymbol{r}, E) / \int_0^{eV_s} N(\boldsymbol{r}, E)dE \qquad \text{(S11)}$$

This is the infamous "setup effect" in which $g(\boldsymbol{r}, V)$ is not proportional to $N(\boldsymbol{r}, E)$. Any setup effect of the $Bi_2Sr_2CaCu_2O_{8+x}$ crystal supermodulation would then be to modulate $\int_0^{eV_s} N(\boldsymbol{r}, E)dE$ periodically at wavevector $\boldsymbol{Q}_S$. One can simulate the effects which this would produce by multiplying a typical $g(\boldsymbol{r}, V)$ by a periodic function $A\mathrm{Cos}(\boldsymbol{Q}_S.\boldsymbol{r})$ and this we have done to generate simulated data in Fig. S3j. Hence, if a setup effect were the predominant phenomenon, the measured $g(\boldsymbol{r}, V)$ data in Fig. S3g would correspond to the image of setup-effect simulated $g(\boldsymbol{r}, V)$ data in Fig S3j. Inspection shows them be quite different, most obviously in that there is virtually no modulation in measured $g(\boldsymbol{r}, V)$ at large positive bias voltage, as would be required to occur if the setup-effect prevails. This analysis rules out the setup effect (Eq. S11) as the predominant cause of modulations at $\boldsymbol{Q}_S$ in all $g(\boldsymbol{r}, V)$ data studied herein.

Hence, variations in the energy separation $\mathcal{E}$ between the bands can be estimated by measuring the energy range between these edges at a constant differential

conductance G as shown, for example, in Fig. 3c of the main text. In our studies we choose to use $G \approx 20$ pS for several practical reasons explained below, but emphasize that the demonstration that $\mathcal{E}(\boldsymbol{r})$ varies periodically with the crystal supermodulation is not contingent on any specific value for G. For example, in Fig. S4 shows $\mathcal{E}(G, \boldsymbol{r})$ maps extracted at selected values of G ranging from 20 pS to 80 pS. One can see that the spatial structure of $\mathcal{E}(G, \boldsymbol{r})$ is extremely similar for 20 pS $< G <$ 80 pS. Moreover, the power spectral density Fourier transform $\mathcal{E}(G, \boldsymbol{q})$ of every one exhibits strong peaks at wavevectors $\pm\boldsymbol{Q_S}$. This means that the relationship between $\mathcal{E}$ and Φ will be qualitatively the same for any G considered here, but with somewhat different amplitudes in $\mathcal{E}$ variations. One additional complication for the $\mathcal{E}(G, \boldsymbol{r})$ measured in the range 40 pS $<$ G $<$ 80 pS and that at $G = 20$ pS is the presence of dark regions at each oxygen dopant ion where strong local maxima occur in $g(\boldsymbol{r}, V)$ at conductance higher than 20 pS, preventing accurate measurement of $\mathcal{E}(\Phi)$. This is another reason we focus on analysis of the $G = 20$ pS charge transfer energy maps so as to avoid the dopant-atom complication. Incidentally, we demonstrate directly in Figs S3b,c; S3e,f; S3h,i; S3k,l that the crystal supermodulation effects on $\mathcal{E}$ occur in regions where dopant ions are absent, ruling them out as a possible contribution to the phenomena under study.

Finally, Fig. S5 (a-c) show $\mathcal{E}(\boldsymbol{r})$ obtained by this $G = 20$ pS procedure in three disjoint fields of view. Each has an area of $19.5 \times 19.5\,\mathrm{nm}^2$ and their $\mathcal{E}(\boldsymbol{r})$ have extremely similar spatial structure, with spatially-averaged values $\langle\mathcal{E}\rangle \approx$ 1.195 eV, 1.205 eV and 1.215 eV respectively. These average charge-transfer energies are in excellent quantitative agreement with those derived from a variety of experimental techniques, including SISTM (27), ARPES (*56*), and optical spectroscopy (*57*) of $Bi_2Sr_2CaCu_2O_{8+x}$, meaning that the choice of $G = 20$ pS at which to measure $\mathcal{E}$ is both practical and quantitatively valid. To further underscore the statistical validity of $\mathcal{E}$, Fig. S5 d,e,f show the phase-averaged, Fourier-filtered $\mathcal{E}(\Phi)$ in the same three fields of view. It is readily apparent that the peak-to-peak amplitude is the same for all three fields of view, and $\mathcal{E}(\Phi)$ is minimized at $\Phi \approx \pi$ in all three fields of view. Ultimately, it is this band-separation at $G = 20$ pS analysis technique which yields the $\mathcal{E}(\Phi)$ that we study throughout the main text.

(IV) Electron-pair Density Visualization $\boldsymbol{n}_{\mathrm{P}}(\boldsymbol{r})$ by SJTM

The macroscopic wavefunctions of the tip and sample forming a Josephson junction can be written as

$$\psi_{\mathrm{T}} = \langle c_{\uparrow}c_{\downarrow}\rangle_T \mathrm{e}^{-\mathrm{i}\varphi_{\mathrm{T}}} = \sqrt{n_T}\mathrm{e}^{-\mathrm{i}\varphi_{\mathrm{T}}} \tag{S12}$$

$$\psi_{\mathrm{S}} = \langle c_{\uparrow}c_{\downarrow}\rangle_P \mathrm{e}^{-\mathrm{i}\varphi_{\mathrm{S}}} = \sqrt{n_P}\mathrm{e}^{-\mathrm{i}\varphi_{\mathrm{S}}} \tag{S13}$$

where n_T (n_P) and φ_T (φ_S) are the electron-pair densities and the phases of the tip (sample), respectively. The product of the Josephson critical current I_J with the junction's normal-state resistance R_N is (*58,59*):

$$I_J R_N \propto \langle c_{\uparrow}c_{\downarrow}\rangle_P \langle c_{\uparrow}c_{\downarrow}\rangle_T \propto \sqrt{n_P}\sqrt{n_T}. \tag{S14}$$

Assuming n_T to be constant, the sample electron-pair density n_P can be measured as

$n_P \propto (I_J R_N)^2$. In a phase-diffusive Josephson junction, the current – voltage $I_P(V_J)$ characteristic is described by the following equation with the maximum Josephson current ($I_m = \frac{I_J^2 Z}{4V_c}$) appearing at non-zero junction voltages V_c:

$$I_P(V_J) = \frac{1}{2} I_J^2 Z\, V_J / (V_J^2 + V_c^2) \quad \text{(S15)}$$

Here, Z is the impedance relevant to re-trapping of the diffusing phase. The first derivative of $I_P(V_J)$ at zero bias is

$$g_0 \equiv \left.\frac{dI_P}{dV_J}\right|_{V_J=0} = \frac{I_J^2 Z}{2{V_c}^2} \propto I_m \quad \text{(S16)}$$

In scanned Josephson tunneling microscopy (SJTM) using superconductive STM tips, the sample electron-pair density can therefore be visualized (*7,8,9*) by measuring either $I_m(\boldsymbol{r})$ or $g_0(\boldsymbol{r})$ and also separately measuring ${R_N}^2(\boldsymbol{r})$ in the identical field of view yielding the electron-pair density as:

$$n_P(\boldsymbol{r}) \propto I_m {R_N}^2(\boldsymbol{r}) \propto g_0 R_N^2(\boldsymbol{r}). \quad \text{(S17)}$$

For these studies, we use a $Bi_2Sr_2CaCu_2O_{8+x}$ nanoflake tip (*7,60*). A single crystal sample of $Bi_2Sr_2CaCu_2O_{8+x}$ at the hole density p = 0.17 ± 0.01 and superconducting transition temperature T_c = 91 K is cleaved in cryogenic ultra-high vacuum. The nanoflake tip is then created by picking up a nanometer scale $Bi_2Sr_2CaCu_2O_{8+x}$ flake from the sample surface. A typical resulting topography is shown in Fig. S2 in which both the locations of individual Bi atoms at the BiO termination layer and the crystal supermodulation are all well resolved (*7,60*).

To extract the phase-diffusive Josephson differential conductance at zero bias g_0 from $g(V_J, \boldsymbol{r})$ we fit this Josephson junction differential conductance spectrum to the 1st derivative of Eq. S15 with free fitting parameters being $\frac{1}{2} I_J^2 Z$, V_c and an additional constant C added to take account of any small conductance offsets that may occur systematically, and from which fits the $g_0(\boldsymbol{r})$ and $I_m(\boldsymbol{r})$ are derived.

Fig. S10 shows a linecut of I_m along the $q = \boldsymbol{Q}_{SM}$ direction, normalized to the background value $I_m(\boldsymbol{q} = \boldsymbol{0})$ for three different experiments in three different fields of view along with the equivalent linecut for g_0 used to generate Fig. 5 of the main text. The consistency in the amplitude of supermodulation in I_m and g_0 across the various experiments demonstrates the repeatability of the superfluid density modulation measurements.

(V) Determination of Equivalent Normal State Junction Resistance R_N

The Josephson tunnel junction resistance $R_N(\boldsymbol{r})$ is difficult to determine in a single $g(\boldsymbol{r},V)$ map because $I_P(V_J)$ is measured in the 50 μV range (Fig. 3b), whereas $R_N(\boldsymbol{r})$ is only quantifiable using differential conductance measurements at the 100 mV range (at $E \gg \Delta_{SIS}$ where for cuprates $\Delta_{SIS} > 50$meV). Hence we use a two-step procedure in which we measure a low voltage range $g_1(\boldsymbol{r},V)$ and a high voltage range $g_2(\boldsymbol{r},V)$ in exactly the same FOV (Fig. S6). The $g_1(\boldsymbol{r},V)$ ranges from-15 mV $< V <$ 15 mV with a setpoint $I_{s1} = 100$ pA , $V_{s1} = 15$mV, while $g_2(\boldsymbol{r},V)$ is acquired from -350 mV $< V <$ 350 mV in the almost identical FOV at setpoint $I_{s2} = 350$ pA , V_{s2} =350 mV so that $V_{s2} \gg \Delta_{SIS}$.

Thus, $g_1(\boldsymbol{r},V)$ and $g_2(\boldsymbol{r},V)$ are obtained in different measurements but nominally the same field of view (FOV). These images, along with their associated topographs $T(\boldsymbol{r})$, are then registered to the exact same FOV by a series of transformations. Each experimental image $T(\boldsymbol{r})$ can be registered to the expected periodic lattice using the Lawlor-Fujita (LF) procedure (*61*), which utilizes a 2D lock-in method to solve for the displacement field $\boldsymbol{r} - \tilde{\boldsymbol{r}}$ between the experimentally measured $T(\boldsymbol{r})$ and a perfectly lattice periodic map $T^0(\boldsymbol{r})$. After this procedure, the lattice is almost exactly periodic, but can sometimes appear sheared from expected $C4$ symmetry. To correct this, we apply a shear transformation to the experimental data. Once the experimental images have been corrected, they are then atomically registered to the perfectly identical FOV by rigidly shifting the maps relative to one another. After this procedure, the $g_1(\boldsymbol{r},V)$ and $g_2(\boldsymbol{r},V)$ data are now in a precisely identical FOV.

The $g_1(\boldsymbol{r},V_J)$ is then fit with a parabolic curve $g_1 = a_1V_J^2 + b_1$over the voltage range $[-V_{s1},V_{s1}]$ at every $\boldsymbol{r}$. A small energy range within which Josephson electron-pair tunneling dominates surrounding $V_J = 0$ is omitted. Next $g_2(\boldsymbol{r},V_J)$ is smoothed and fit with a parabolic curve $g_2 = a_2V_J^2 + b_2$ over a voltage range $[-V_0,V_0]$, where $V_{s1} < V_0 < \Delta_{SIS}$. Finally, we determine the scaling factors between $g_2(\boldsymbol{r})$ and $g_1(\boldsymbol{r})$ via $g_1(\boldsymbol{r}) = g_2'(\boldsymbol{r}) = \alpha(\boldsymbol{r})g_2(\boldsymbol{r}) + \beta(\boldsymbol{r})$, where $\alpha = \frac{a_1}{a_2}$, $\beta = b_1 - b_2\frac{a_1}{a_2}$.

$$g_2'(\boldsymbol{r},V_J) = \alpha(\boldsymbol{r})g_2(\boldsymbol{r},V_J) + \beta(\boldsymbol{r}). \quad \text{(S18)}$$

The rescaled $g_2'(V_J)$ and the original $g_1(V_J)$ when plotted on the same graph are now virtually indistinguishable (Fig. S6c), and the high-voltage conductance $g_2'(\boldsymbol{r},V_J)$ associated with the junction resistance R_N for the conditions of Josephson tunneling be read off for each $\boldsymbol{r}$. The rescaled $g_2'(\boldsymbol{r},V)$ and the original $g_1(\boldsymbol{r},V)$ images are shown in Fig. S7 demonstrating the fidelity of the rescaling process. Finally, we can establish the normal state junction resistance corresponding to the low-voltage $g_1(\boldsymbol{r},V_J)$ map:

$$R_N(\boldsymbol{r}) \equiv \frac{1}{g_2'(r,V_{s2})}. \quad \text{(S19)}$$

A typical example of such data $R_N(\boldsymbol{r})$ which is used to establish $n_P(\boldsymbol{r}) \propto g_0R_N^2(\boldsymbol{r})$ as in Figs 4,5 is shown in Fig. S8.

(VI) Determination of the Relationships $\tilde{\mathcal{E}}(\Phi)$ and $\tilde{n}_P(\Phi)$

To measure the dependence of charge transfer energy modulations $\mathcal{E}(\Phi)$ and electron – pair density modulations $n_P(\Phi)$ on the supermodulation phase Φ, the first step is to Fourier filter the quantity of interest $A(\boldsymbol{r})$, either $\mathcal{E}(\boldsymbol{r})$ or $n_P(\boldsymbol{r})$, retaining only contributions from the wavevectors close to $\boldsymbol{q} = \pm\boldsymbol{Q}_S$ and adding the constant background $\langle A\rangle$:

$$\tilde{A}(\boldsymbol{r}) = \langle A\rangle + \frac{1}{\sigma\sqrt{2\pi}}\int A(\boldsymbol{q})e^{-i\boldsymbol{q}\cdot\boldsymbol{r}}\left(e^{\frac{|q-Q_S|^2}{2\sigma^2}} + e^{\frac{|q+Q_S|^2}{2\sigma^2}}\right)\mathrm{d}\boldsymbol{q}. \tag{S20}$$

Next, we calculate the supermodulation phase $\Phi(\boldsymbol{r})$ from the topograph $T(\boldsymbol{r})$ in the same FOV as $A(\boldsymbol{r})$. We then bin each pixel in the FOV according to its local phase $\Phi(\boldsymbol{r})$. Since each pixel is associated with a pair $\tilde{A}(\boldsymbol{r})$: $\Phi(\boldsymbol{r})$, this yields a two-dimensional histogram showing the coincidence of these values. This is shown for $\tilde{\mathcal{E}}(\boldsymbol{r})$ and $\tilde{n}_P(\boldsymbol{r})$ in Fig. S9a and S9c, which are the core empirical data for this project. The ultimate step is to average $\tilde{A}(\boldsymbol{r})$ for every $\boldsymbol{r}$ with a given value of Φ yielding the plot $\tilde{A}(\Phi)$ as shown for $\tilde{\mathcal{E}}(\boldsymbol{r})$ and $\tilde{n}_P(\boldsymbol{r})$ in Fig. S9b, d.

For $Bi_2Sr_2CaCu_2O_{8+x}$ a similar procedure has been used to plot the energy gap (colloquially in cuprate studies the "pseudogap") for single-particle excitations $\Delta(\Phi)$ as a function of phase (*25*). The results are that Δ is also modulated at $\boldsymbol{Q_S}$ and that its value is minimized at $\Phi \approx \pi$. Therefore, the data in Fig. 5 imply Δ and n_p are out of phase by π for $Bi_2Sr_2CaCu_2O_{8+x}$. This is as would be expected in a strongly correlated unconventional superconductor described by the Hubbard model. In that case, it is well known that the pseudogap to single-particle excitations evolves oppositely (*38,39*) to the amplitude of the amplitude of electron-pair condensate Ψ.

(VII) Estimation of α from Three-band CuO_2 Hubbard Model Calculations

Theoretical calculations of Ψ and $\mathcal{E}$ have been carried out by numerical solution of the Emery three-band model within the framework of cluster dynamical mean field theory (CDMFT). One can extract predicted values of $\alpha = dn_P/d\mathcal{E}$ for comparison with the experimental results in Fig. 5d. Here the measured $g_0R_N^2$ is proportional to n_P, but the constants of proportionality are unknown so one we cannot (yet) compare this quantity to $n_P = |\Psi|^2$ calculated using CDMFT in absolute units. Thus, one needs to normalize both the experimental results and the theoretical predictions in a consistent manner.

To do so, we first normalize measured $g_0R_N^2$ to its mean value in the experimental FOV:

$$\bar{n}_P = \frac{g_0R_N^2}{\langle g_0R_N^2\rangle} \tag{S21}$$

as shown in Fig. 5. The CDMFT calculated $|\Psi|$ in Ref. (*12)* is normalized to the reference value of $|\Psi|$ for the so-called covalent case $|\Psi|_{\mathrm{cov}} = 0.0774$, whose input material parameters are chosen to be representative of $Bi_2Sr_2CaCu_2O_{8+x}$.

$$\left|\overline{\langle c_{\uparrow} c_{\downarrow}\rangle}\right|^{\mathrm{BSCCO}} = \frac{|\Psi|}{|\Psi|_{\mathrm{cov}}} \tag{S22}$$

Normalization of results for La_2CuO_4 in Ref. (*16*) is achieved similarly as

$$\left|\overline{\langle c_{\uparrow} c_{\downarrow}\rangle}\right|^{\mathrm{LSCO}} = \frac{|\Psi|}{|\Psi|_{\mathrm{LSCO}}}, \tag{S23}$$

where $|\Psi|_{\mathrm{LSCO}} = 0.0154$. Since $Bi_2Sr_2CaCu_2O_{8+x}$ is among the cuprates with the smallest value of $\mathcal{E}$ and La_2CuO_4 is among those with the largest, we define a range of α for the cuprates ranging between the values of Ref. (*12*) and Ref. (*16*) to arrive at the approximate inequality $0.3 \lesssim \alpha \lesssim 1.0\ \mathrm{eV}^{-1}$. This is represented by the yellow shaded wedge in Fig. 5d. The measured value of $\alpha = -0.81 \pm 0.17\ \mathrm{eV}^{-1}$ falls in this range and is very close to the DMFT prediction for $Bi_2Sr_2CaCu_2O_{8+x}$.

Supplementary References

46 Y. Tokura, S. Koshihara, T. Arima, H. Takagi, S. Ishibashi, T. Ido, S. Uchida, Cu-O network dependence of optical charge-transfer gaps and spin-pair excitations in single-CuO_2-layer compounds. *Phys. Rev. B* ***41***, 11657(R) (1990).

47 S. L. Cooper, G. A. Thomas, A. J. Millis, P. E. Sulewski, J. Orenstein, D. H. Rapkine, S-W. Cheong, P. L. Trevor, Optical studies of gap, exchange, and hopping energies in the insulating cuprates. *Phys. Rev. B* ***42***, 10785(R) (1990).

48 M. C. Aronson, S. B. Dierker, B. S. Dennis, S-W. Cheong, Z. Fisk, Pressure dependence of the superexchange interaction in antiferromagnetic La_2CuO_4. *Phys Rev B* ***44,*** 4657 (1991).

49 S.-L. Yang, J. A. Sobota, Y. He, Y. Wang, D. Leuenberger, H. Soifer, M. Hashimoto, D. H. Lu, H. Eisaki, B. Moritz, T. P. Devereaux, P. S. Kirchmann, Z.-X. Shen, Revealing the Coulomb interaction strength in a cuprate superconductor**.** *Phys Rev B* ***96***, 245112 (2017).

50 M. Le Tacon, G. Ghiringhelli, J. Chaloupka, M. Moretti Sala, V. Hinkov, M. W. Haverkort, M. Minola, M. Bakr, K. J. Zhou, S. Blanco-Canosa, C. Monney, Y. T. Song, G. L. Sun, C. T. Lin, G. M. De Luca, M. Salluzzo, G. Khaliullin, T. Schmitt, L. Braicovich, B. Keimer, Intense paramagnon excitations in a large family of high-temperature superconductors. *Nat. Phys.* ***7*****,** 725–730 (2011).

51 Y. Y. Peng, G. Dellea, M. Minola, M. Conni, A. Amorese, D. Di Castro, G. M. De Luca, K. Kummer, M. Salluzzo, X. Sun, X. J. Zhou, G. Balestrino, M. Le Tacon, B. Keimer, L. Braicovich, N. B. Brookes, G. Ghiringhelli*,* Influence of apical oxygen on the extent of in-plane exchange interaction in cuprate superconductors. *Nat. Phys.* ***13***, 1201-1206 (2017).

52 G. Levy, M. Yaari, T. Z. Regier, A. Keren, Experimental determination of superexchange energy from two-hole spectra. https://arxiv.org/abs/2107.09181 (2021).

53 D. Grebille, H. Leligny, A. Ruyter, P. Labbé, B. Raveau, Static disorder in the incommensurate structure of the high T_c superconductor $Bi_2Sr_2CaCu_2O_{8+\delta}$. *Acta Crystallogr. Sect. B Struct. Sci.* ***52***, 628–642 (1996).

54 K. McElroy, Jinho Lee, J. Slezak, D. Lee, H. Eisaki, S. Uchida, J. C. Davis, Atomic-scale sources and mechanism of the nanoscale electronic disorder in $Bi_2Sr_2CaCu_2O_{8+\delta}$. *Science* ***309***, 1048-1052 (2005).

55 Y. Kohsaka, T. Hanaguri, M. Azuma, M. Takano, J. C. Davis, H. Takagi, Visualization of the emergence of the pseudogap state and the evolution to superconductivity in a lightly hole-doped Mott insulator. *Nat. Phys.* ***8***, 534-538 (2012).

56 S.-L. Yang, J. A. Sobota, Y. He, Y. Wang, D. Leuenberger, H. Soifer, M. Hashimoto, D. H. Lu, H. Eisaki, B. Moritz, T. P. Devereaux, P. S. Kirchmann, Z.-X. Shen, Revealing the Coulomb interaction strength in a cuprate superconductor**.** *Phys Rev B* ***96***, 245112 (2017).

57 T. Itoh, K. Fueki, Y. Tanaka, H. Ihara, Optical conductivity spectra and electronic structure. of $Bi_2Sr_2(Y_{1-x}Ca_x)Cu_2O_y$ system. *J. Phys. Chem. Solids* ***60***, 41-51 (1999).

58 H. Kimura, R. P. Barber, Jr., S. Ono, Y. Ando, R. C. Dynes, Josephson scanning tunneling microscopy: A local and direct probe of the superconducting order parameter. *Phys. Rev. B* ***80***, 144506 (2009).

59 H. Kimura, R. P. Barber, Jr., S. Ono, Y. Ando, R. C. Dynes, Scanning Josephson tunneling microscopy of single-crystal $Bi_2Sr_2CaCu_2O_{8+\delta}$ with a conventional superconducting tip. *Phys. Rev. Lett.* ***101***, 037002 (2008).

60 Z. Du, H. Li, S. H. Joo, E. P. Donoway, J. Lee, J. C. S. Davis, G. Gu, P. D. Johnson, K. Fujita, Imaging the energy gap modulations of the cuprate pair-density-wave state. *Nature* ***580***, 65-70 (2020).

61 M. J. Lawler, K. Fujita, J. Lee, A. R. Schmidt, Y. Kohsaka, C. K. Kim, H. Eisaki, S. Uchida, J. C. Davis, J. P. Sethna, E-A. Kim, Intra-unit-cell electronic nematicity of the high-T_c copper-oxide pseudogap states. *Nature* ***466***, 347 – 351 (2010).

Supplementary Figures

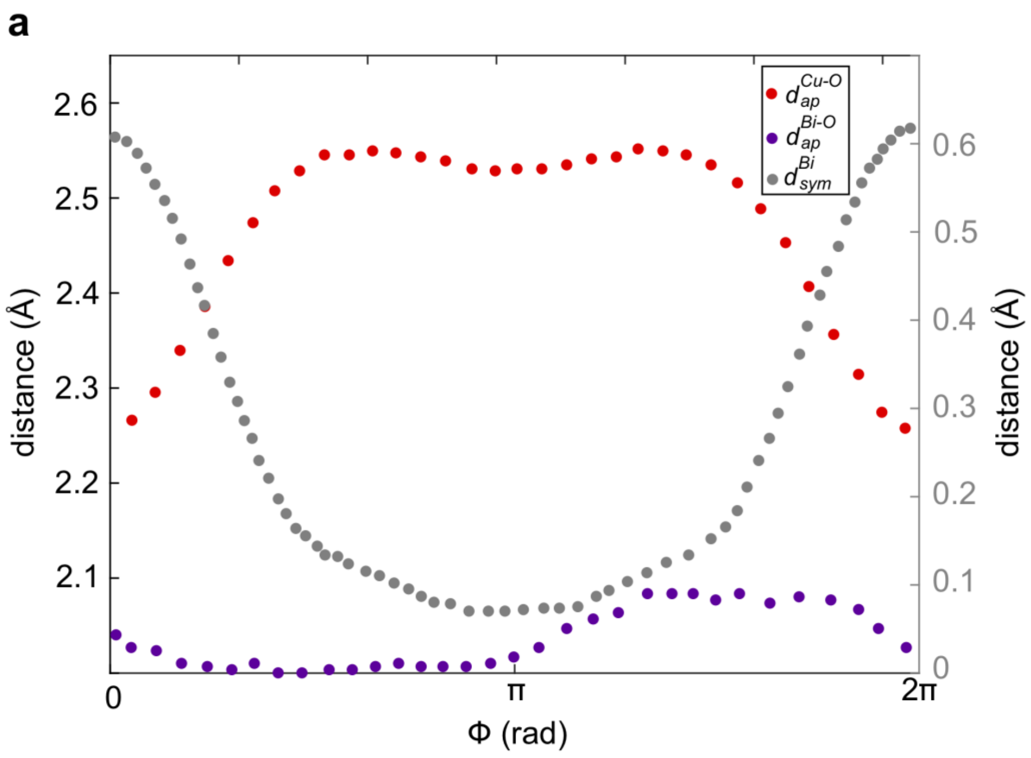

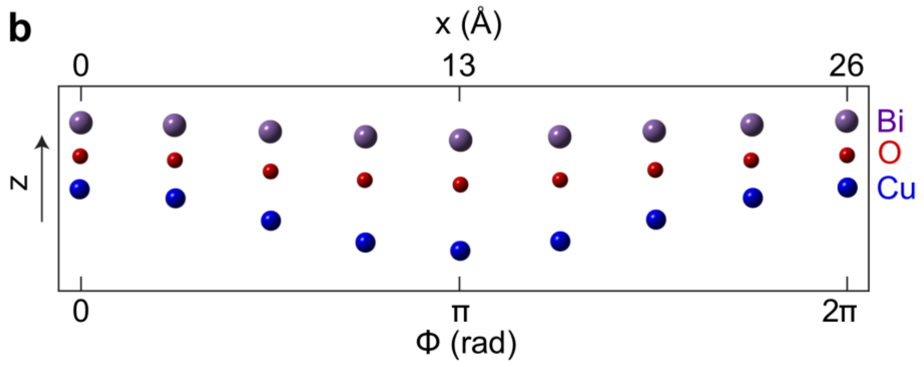

SM Figure 1: $Bi_2Sr_2CaCu_2O_{8+x}$ Crystal Supermodulation

a. Left hand axis: apical Cu-O and Bi-O distances as a function of supermodulation phase Φ from Ref. 53. Right hand axis: displacement of Bi-O termination layer from symmetry plane of crystal as a function of supermodulation phase Φ from X-ray data. The latter measures the same quantity as the STM topograph $T(\boldsymbol{r})$ enabling one to form a one-to-one mapping between the supermodulation phase Φ we extract from $T(\boldsymbol{r})$ and the phase variable in the X-ray refinement. Further, the relatively small amplitude of Bi-O modulation compared to the Cu-O apical modulation enables one to make a direct connection between Φ and δ.

b. Schematic showing the atomic modulations induced along the z axis by the supermodulation. The CuO_2 layer modulates with a larger amplitude than the SrO layer, resulting in δ being maximal at $\Phi = \pi$, where the topograph elevation has its minimum.

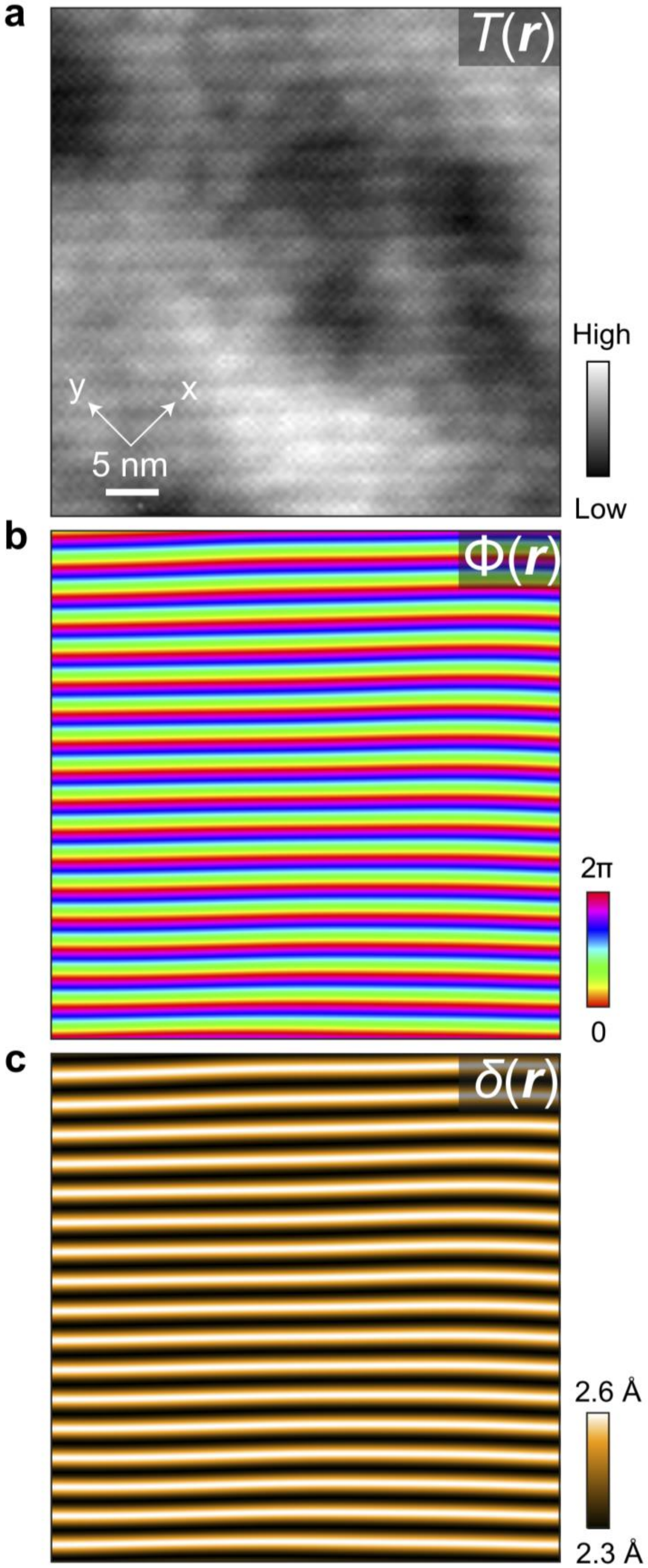
a
T(r)
High
Low
y
x
5 nm
b
Φ(r)
2π
0
c
δ(r)
2.6 Å
2.3 Å

SM Figure 2: $Bi_2Sr_2CaCu_2O_{8+x}$ Nanoflake Topography

Evaluation of a: $T(\boldsymbol{r})$, b: $\Phi(\boldsymbol{r})$ and c: $\delta(\boldsymbol{r})$ when using the $Bi_2Sr_2CaCu_2O_{8+x}$ nanoflake tip (*7,60*). The atomic structure of the Bi-O termination layer and the supermodulation are clearly visible in $T(\boldsymbol{r})$. The supermodulation phase can be readily extracted from the total $T(\boldsymbol{r})$ signal and is related to $\delta(\boldsymbol{r})$ through the fitting in Eq. S7.

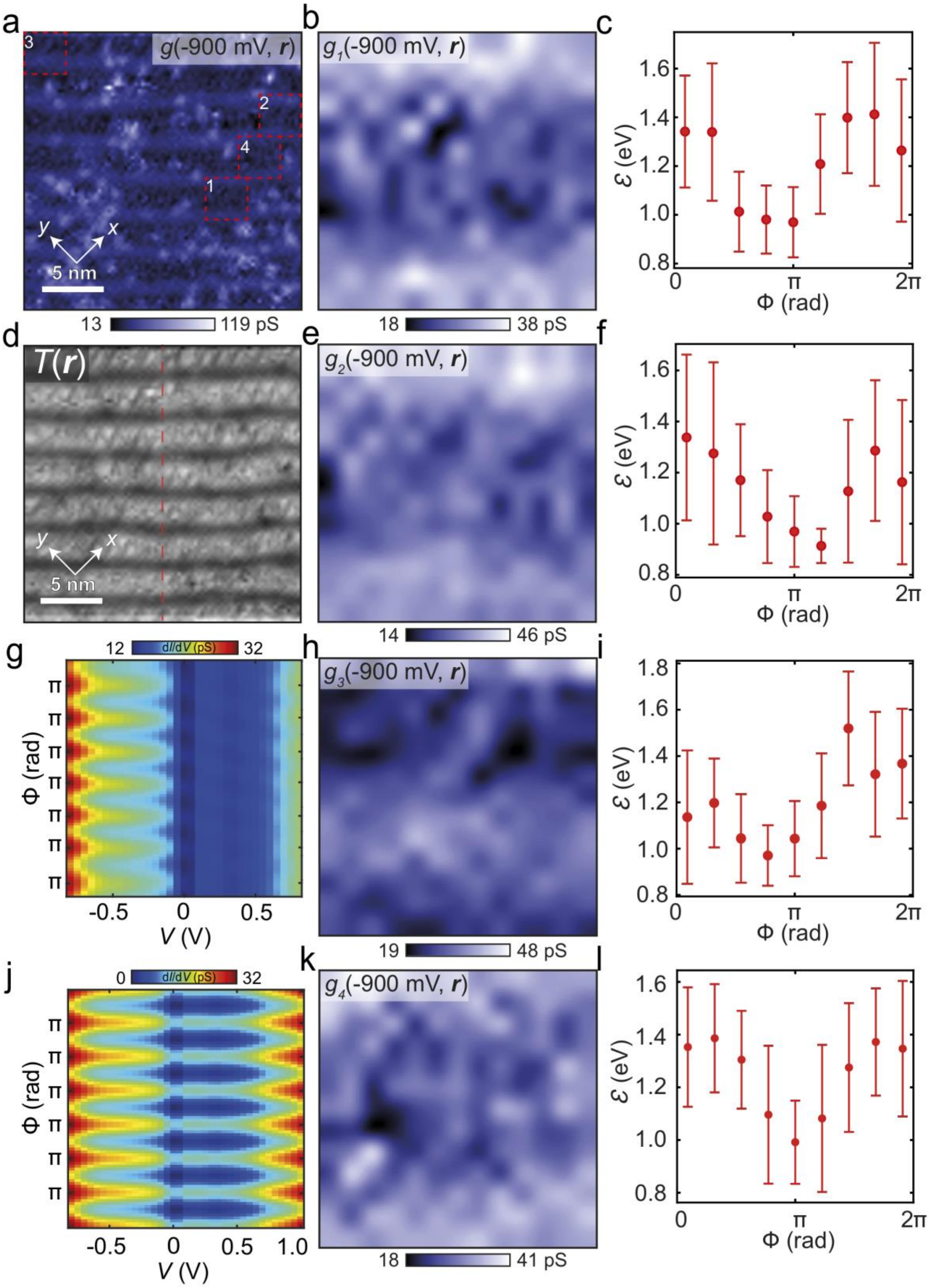

a
g(-900 mV, r)
3
2
4
1
y
x
5 nm
13
119 pS
b
g1(-900 mV, r)
18
38 pS
c
ε (eV)
Φ (rad)
d
T(r)
5 nm
e
g2(-900 mV, r)
14
46 pS
f
g
12
dI/dV (pS)
32
Φ (rad)
V (V)
h
g3(-900 mV, r)
19
48 pS
i
j
0
dI/dV (pS)
32
k
g4(-900 mV, r)
18
41 pS
l

SM Figure 3: Periodic Modulations in High-Voltage $\boldsymbol{g}(\boldsymbol{r},\boldsymbol{V})$ and Low-Voltage $\boldsymbol{I_m}(\boldsymbol{r},\boldsymbol{V})$

a. $g(-900\ \mathrm{mV},\ \boldsymbol{r})$ showing the distribution of O dopants. Four regions without any dopants have been indicated with dashed red boxes.
b. $g(-900\ \mathrm{mV},\ \boldsymbol{r})$ in dopant-free region 1.
c. $\mathcal{E}(\Phi)$ obtained in dopant-free region 1.
d. $T(\boldsymbol{r})$ showing tip-trajectory along a direction perpendicular to the supermodulation as a red dashed line.
e. $g(-900\ \mathrm{mV},\ \boldsymbol{r})$ in dopant-free region 2.
f. $\mathcal{E}(\Phi)$ obtained in dopant-free region 2.
g. Spectrogram of $g(\boldsymbol{r},V)$ along the trajectory indicated by the red dashed line in a. Modulations of the lower filled bands are clearly visible. The upper empty states modulate very weakly.
h. $g(-900\ \mathrm{mV},\ \boldsymbol{r})$ in dopant-free region 3.
i. $\mathcal{E}(\Phi)$ obtained in dopant-free region 3.
j. Simulated spectrogram that would occur due to a setup effect arising from the topograph supermodulation. Both the filled and empty states modulate strongly.
k. $g(-900\ \mathrm{mV},\ \boldsymbol{r})$ in dopant-free region 4.
l. $\mathcal{E}(\Phi)$ obtained in dopant-free region 4.

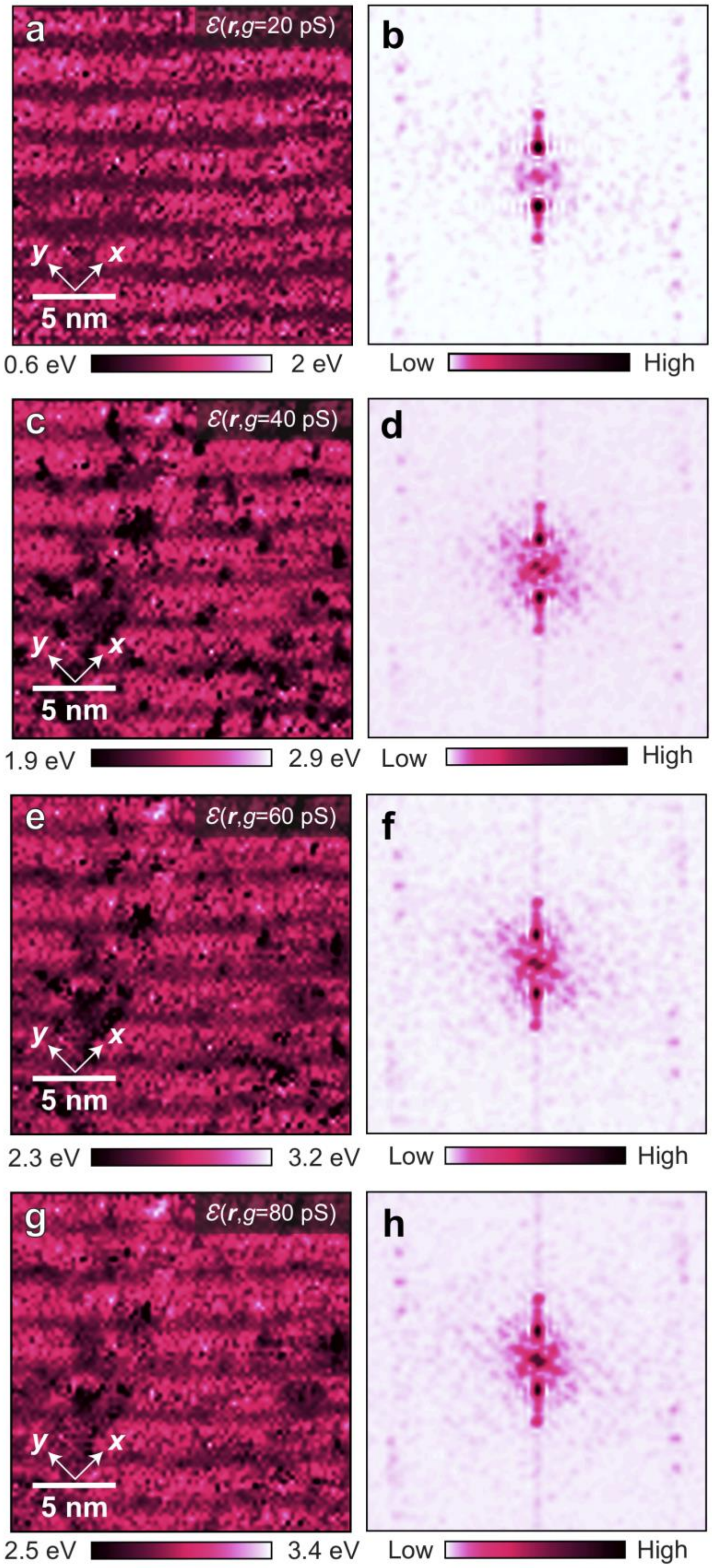

a
$\mathcal{E}(\mathbf{r},g=20\text{ pS})$
y
x
5 nm
0.6 eV
2 eV
b
Low
High
c
$\mathcal{E}(\mathbf{r},g=40\text{ pS})$
y
x
5 nm
1.9 eV
2.9 eV
d
Low
High
e
$\mathcal{E}(\mathbf{r},g=60\text{ pS})$
y
x
5 nm
2.3 eV
3.2 eV
f
Low
High
g
$\mathcal{E}(\mathbf{r},g=80\text{ pS})$
y
x
5 nm
2.5 eV
3.4 eV
h
Low
High

SM Figure 4: Imaging $\mathcal{E}(G, \boldsymbol{r})$ in Different G

Calculated $\mathcal{E}(G, \boldsymbol{r})$ for input differential conductance G of a: $20\ \mathrm{pS}$, c: $40\ \mathrm{pS}$, e: $60\ \mathrm{pS}$ and g: $80\ \mathrm{pS}$ showing that the spatial structure of $\mathcal{E}$ is relatively insensitive to our choice of G, apart from the presence of Oxygen dopant peaks at $G \geq 40\ \mathrm{pS}$. Panels b, d, f and h show the corresponding power spectral densities, which have clear peaks at $\boldsymbol{q} \approx \pm\boldsymbol{Q}_S$ for all values G considered.

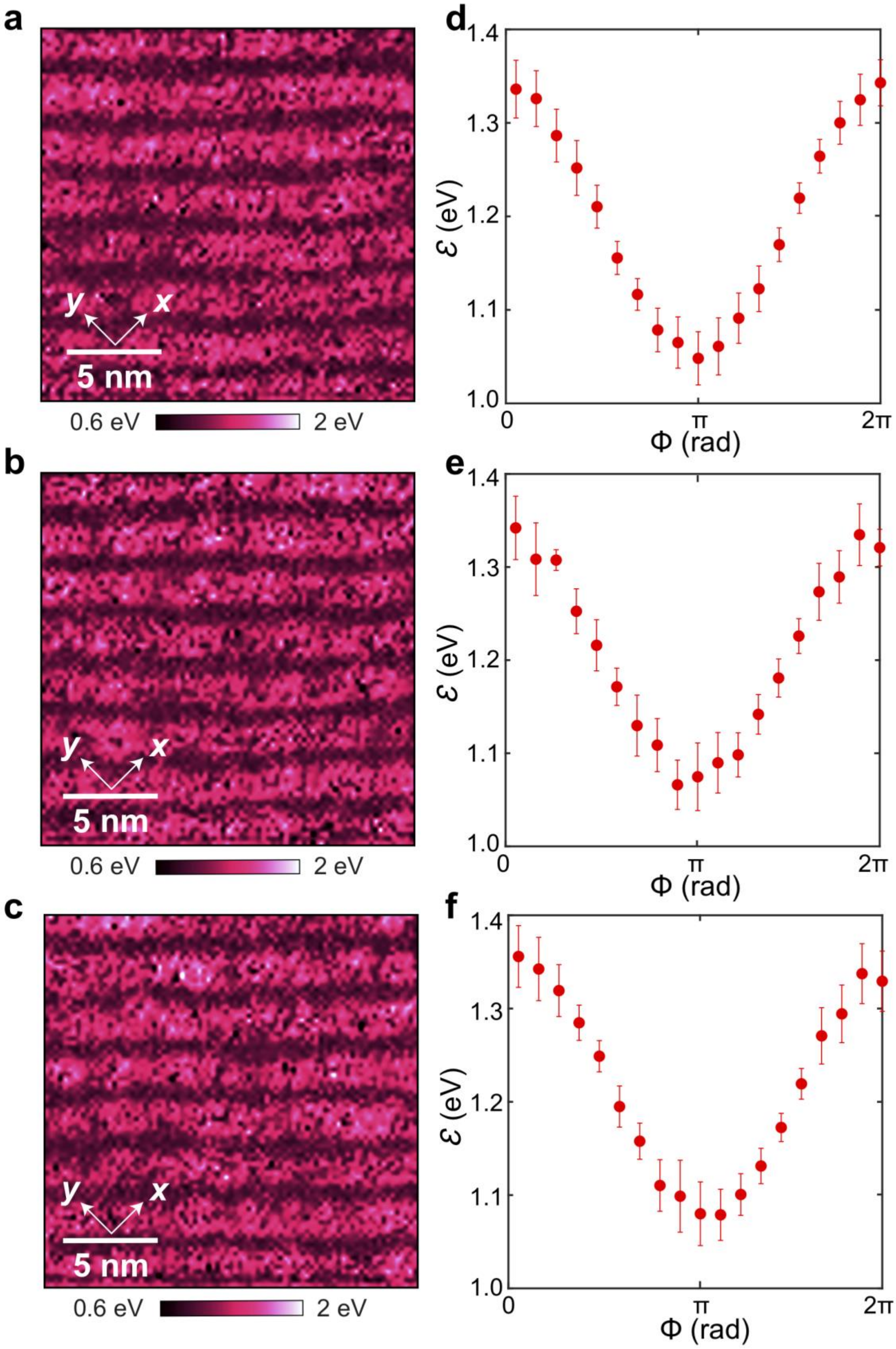
a
y
x
5 nm
0.6 eV
2 eV
b
y
x
5 nm
0.6 eV
2 eV
c
y
x
5 nm
0.6 eV
2 eV
d
1.4
1.3
1.2
1.1
1.0
ℰ (eV)
0
π
2π
Φ (rad)
e
1.4
1.3
1.2
1.1
1.0
ℰ (eV)
0
π
2π
Φ (rad)
f
1.4
1.3
1.2
1.1
1.0
ℰ (eV)
0
π
2π
Φ (rad)

SM Figure 5: Imaging $\mathcal{E}(\boldsymbol{r})$ in Different Fields of View.

(a-c) $\mathcal{E}(\boldsymbol{r})$ calculated in 3 disjoint $19.5\ \times 19.5\ \mathrm{nm}^2$ fields of view.

(d-f) Phase-averaged $\tilde{\mathcal{E}}(\Phi)$ calculated in 3 disjoint $19.5\ \times 19.5\ \mathrm{nm}^2$ fields of view. $\tilde{\mathcal{E}}(\Phi)$ is peaked at $\Phi \approx \pi$ with a peak-to-peak amplitude of ~ 0.3 eV in all three fields of view.

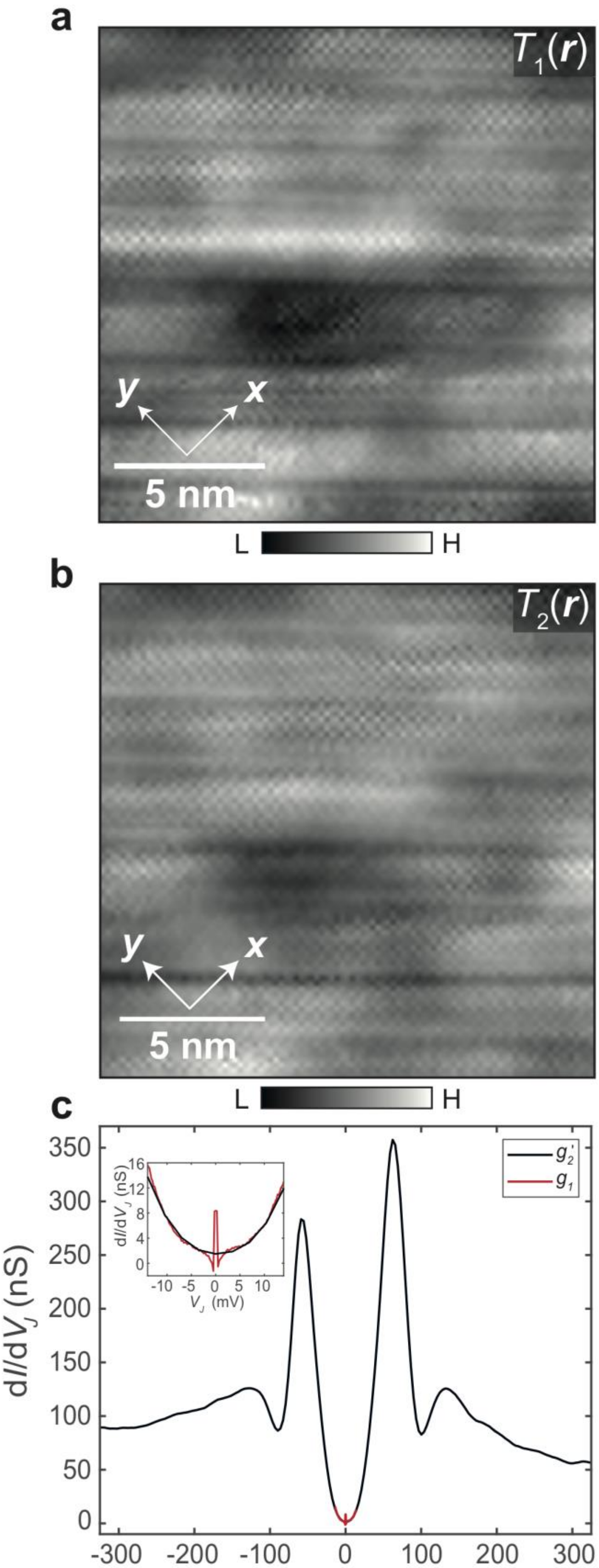
a
$T_1(\boldsymbol{r})$
y
x
5 nm
L
H
b
$T_2(\boldsymbol{r})$
y
x
5 nm
L
H
c
g_2'
g_1
dI/dV_J (nS)
V_J (mV)
350
300
250
200
150
100
50
0
-300
-200
-100
0
100
200
300
16
12
8
4
0
-10
-5
0
5
10

SM Figure 6: Scaling High-Voltage and Low-Voltage $\boldsymbol{g(r, V_J)}$ for $\boldsymbol{R_N(r)}$

(a-b) Simultaneous topographies of $g_1(\boldsymbol{r}, V_J)$ and $g_2(\boldsymbol{r}, V_J)$.

c. Typical example of a rescaled $g_2'(V_J)$ and the original $g_1(V_J)$ spectra. Inset: Same spectra plotted for voltages in the range $-V_{s1} \leq V_J \leq V_{s1}$.

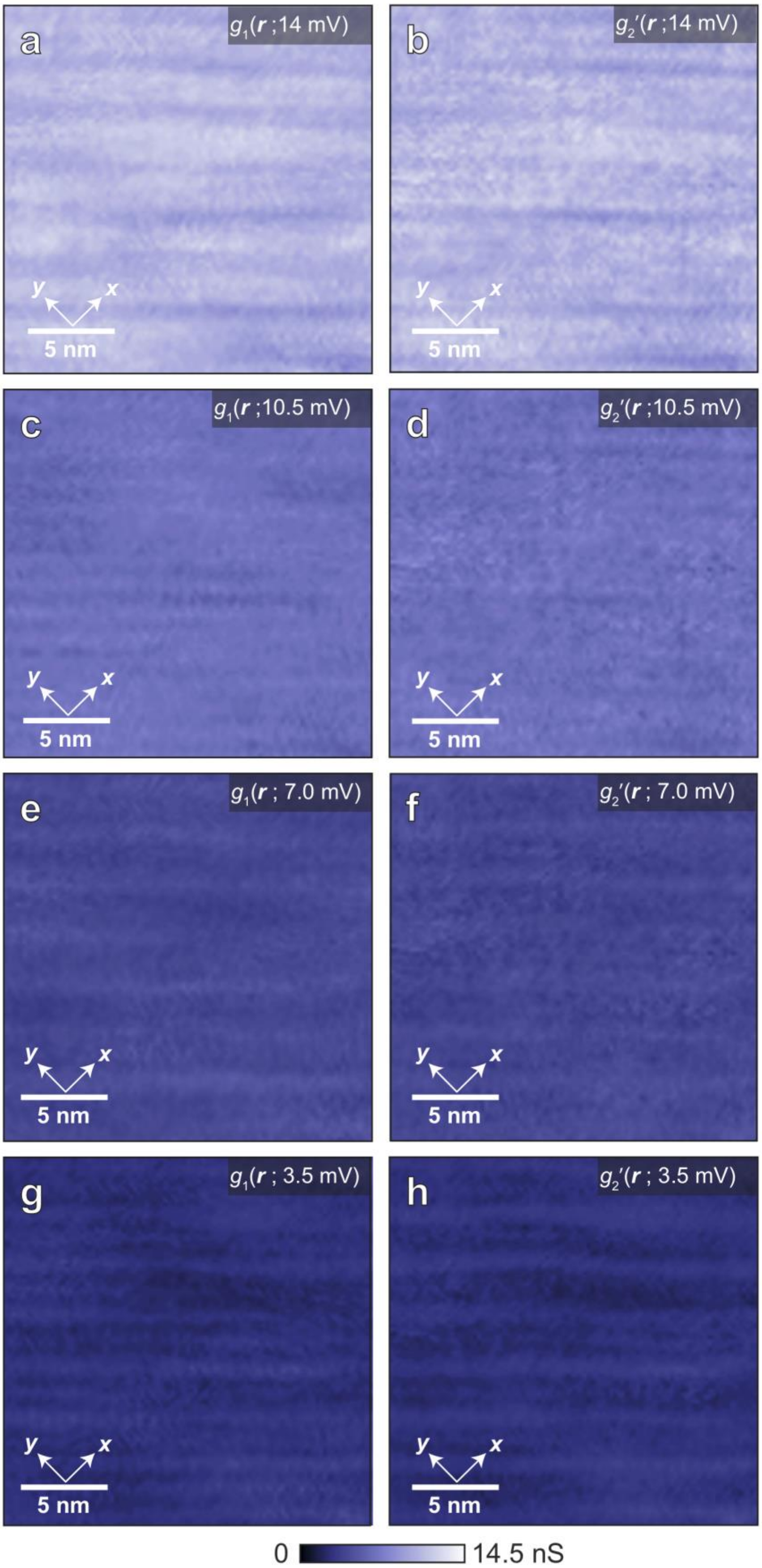
a
$g_1(\boldsymbol{r};14\text{ mV})$
b
$g_2'(\boldsymbol{r};14\text{ mV})$
c
$g_1(\boldsymbol{r};10.5\text{ mV})$
d
$g_2'(\boldsymbol{r};10.5\text{ mV})$
e
$g_1(\boldsymbol{r};7.0\text{ mV})$
f
$g_2'(\boldsymbol{r};7.0\text{ mV})$
g
$g_1(\boldsymbol{r};3.5\text{ mV})$
h
$g_2'(\boldsymbol{r};3.5\text{ mV})$
y
x
5 nm
0
14.5 nS

SM Figure 7: Rescaled $\boldsymbol{g(r,V)}$ Images

(a-h) The rescaled $g_2'(\boldsymbol{r},V)$ and original $g_1(\boldsymbol{r},V)$ images compared at voltages ranging from $3.5\ \mathrm{mV}$ to $14\ \mathrm{mV}$. They are virtually identical, validating the scaling procedure used to obtain $R_N(\boldsymbol{r})$.

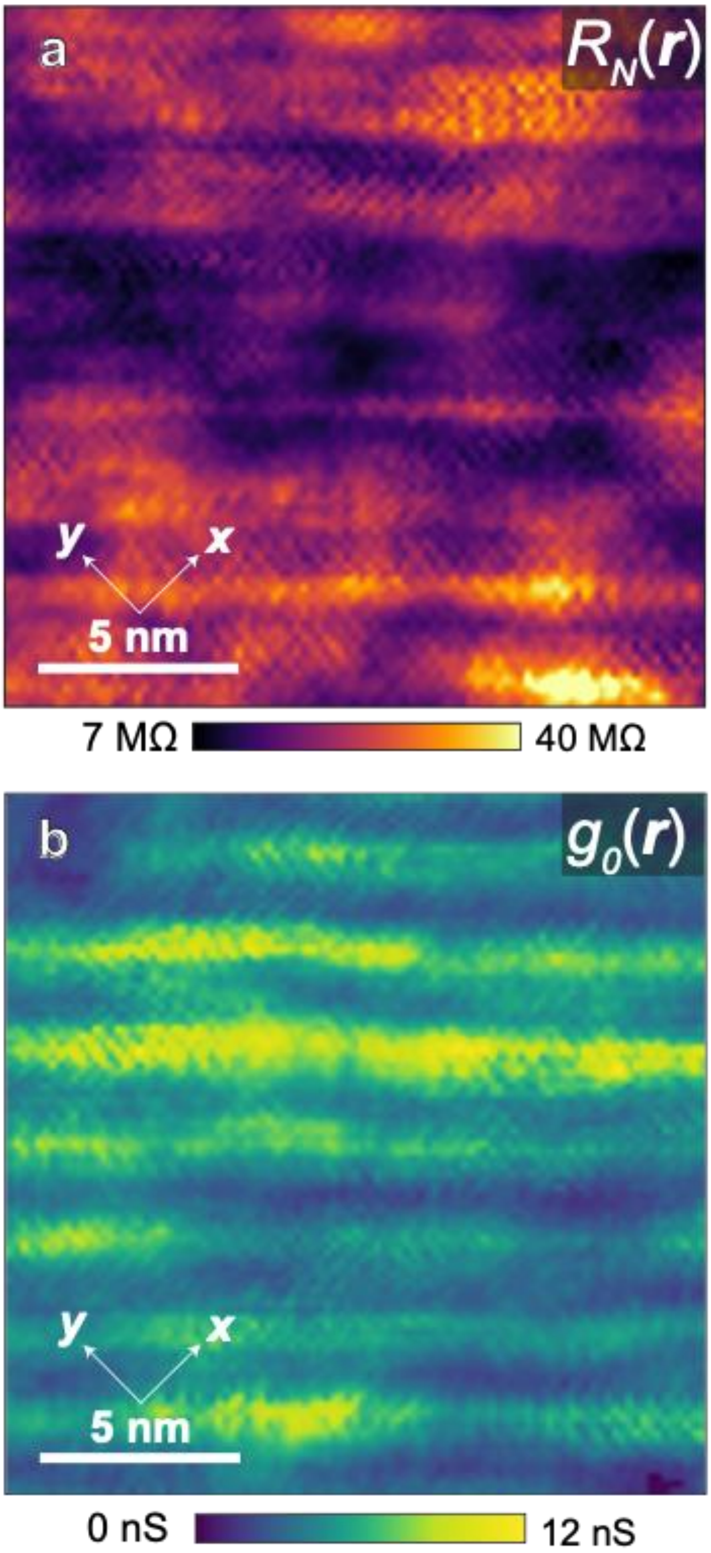
a
$R_N(\boldsymbol{r})$
y
x
5 nm
7 MΩ
40 MΩ
b
$g_0(\boldsymbol{r})$
y
x
5 nm
0 nS
12 nS

SM Figure 8: Imaging $\boldsymbol{R_N(r)}$ and $\boldsymbol{g_0(r)}$

(a) Image of the normal-state junction resistance $R_N(\boldsymbol{r}) \equiv 1/g_2'(\boldsymbol{r}, V_{s2})$ at which the $g_1(\boldsymbol{r}, V_J)$ map was measured.

(b) Image of $g_0(\boldsymbol{r})$ in the same field of view.

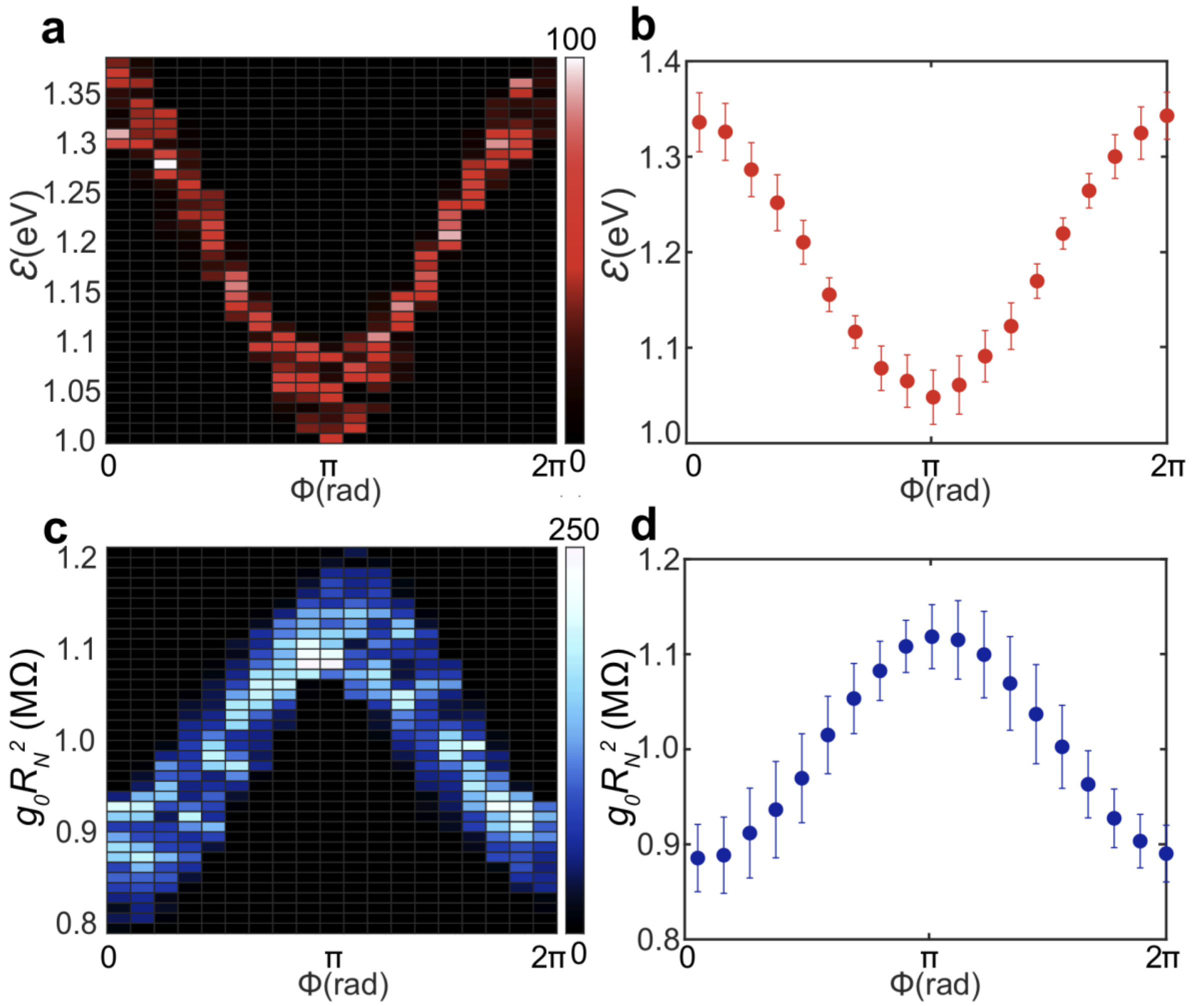
a
b
c
d
100
0
250
0
ℰ(eV)
1.35
1.3
1.25
1.2
1.15
1.1
1.05
1.0
1.4
Φ(rad)
0
π
2π
$g_0R_N^{\ 2}$ (MΩ)
1.2
1.1
1.0
0.9
0.8

SM Figure 9: Determining $\tilde{\mathcal{E}}(\boldsymbol{\Phi})$ and $\tilde{\boldsymbol{n}}_{\boldsymbol{P}}(\boldsymbol{\Phi})$

a. Two-dimensional histogram of $\tilde{\mathcal{E}}$ vs Φ, clearly showing a minimum at $\Phi \approx \pi$.

b. $\tilde{\mathcal{E}}(\Phi)$ obtained by averaging $\tilde{\mathcal{E}}$ within each phase bin.

c. Two-dimensional histogram of $\tilde{n}_P \propto g_0(\boldsymbol{r}) {R_N}^2(\boldsymbol{r})$ vs Φ, clearly showing a maximum at $\Phi \approx \pi$.

d. $\tilde{n}_P(\Phi) \propto g_0 {R_\mathrm{N}}^2(\Phi)$ obtained by averaging $\tilde{n}_P$within each phase bin.

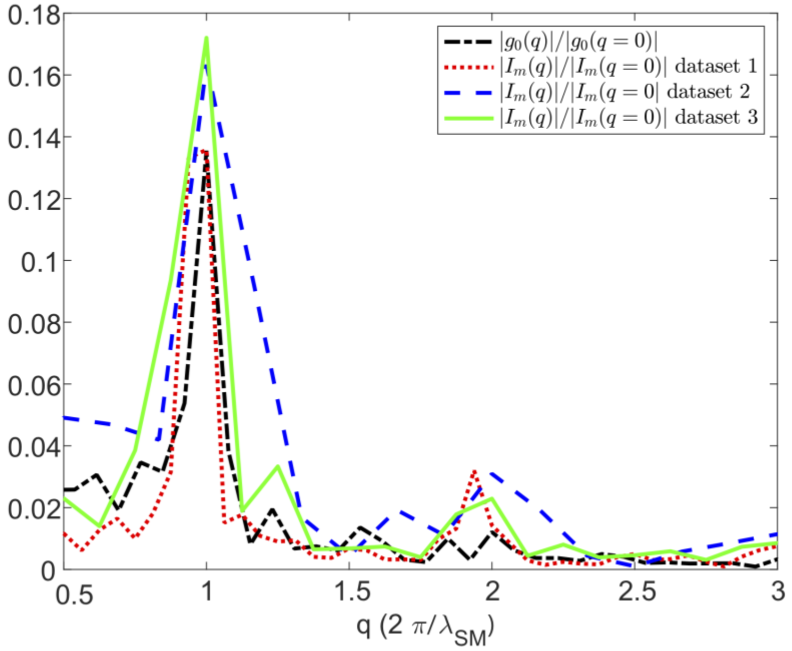

0.18
0.16
0.14
0.12
0.1
0.08
0.06
0.04
0.02
0
0.5
1
1.5
2
2.5
3
q (2 π/λ_SM)
|g_0(q)|/|g_0(q = 0)|
|I_m(q)|/|I_m(q = 0)| dataset 1
|I_m(q)|/|I_m(q = 0| dataset 2
|I_m(q)|/|I_m(q = 0)| dataset 3

SM Figure 10: Repeatability of I_m Supermodulation Amplitude

Fourier transform linecuts of I_m from three different experiments in three different fields of view along with the equivalent linecut for g_0. The black dashed curve corresponds to the g_0 data used to compute electron-pair density n_p in the main text.
